# Endophytic fungi: a reservoir of antibacterials

**DOI:** 10.3389/fmicb.2014.00715

**Published:** 2015-01-08

**Authors:** Sunil K. Deshmukh, Shilpa A. Verekar, Sarita V. Bhave

**Affiliations:** Department of Natural Products, Piramal Enterprises LimitedMumbai, India

**Keywords:** endophytic fungi, antibacterial compounds, natural products, drug resistance, medicinal plants

## Abstract

Multidrug drug resistant bacteria are becoming increasingly problematic particularly in the under developed countries of the world. The most important microorganisms that have seen a geometric rise in numbers are Methicillin resistant *Staphylococcus aureus*, Vancomycin resistant *Enterococcus faecium*, Penicillin resistant *Streptococcus pneumonia* and multiple drug resistant tubercule bacteria to name a just few. New drug scaffolds are essential to tackle this every increasing problem. These scaffolds can be sourced from nature itself. Endophytic fungi are an important reservoir of therapeutically active compounds. This review attempts to present some data relevant to the problem. New, very specific and effective antibiotics are needed but also at an affordable price! A Herculean task for researchers all over the world! In the Asian subcontinent indigenous therapeutics that has been practiced over the centuries such as Ayurveda have been effective as “handed down data” in family generations. May need a second, third and more “in-depth investigations?”

## Introduction

The last two decades have witnessed a rise in the numbers of Methicillin resistant *Staphylococcus aureus* (MRSA), Vancomycin resistant *Enterococcus faecium* (VRE) and Penicillin resistant *Streptococcus pneumoniae* (PRSP) and a variety of antibiotics (Menichetti, 2005). New drugs such as Linezolid and Daptomycin have already acquired resistance (Mutnick et al., 2003; Skiest, 2006). MDR- and XDR-TB (Gillespie, 2002; LoBue, 2009) are emerging global threats, being difficult to diagnose, expensive to treat and with variable results. Rice (2008) reported that the ESKAPE organism's *E. faecium, S. aureus, Klebsiella pneumoniae, Acinetobacter baumanii, P. aeruginosa*, and *Enterobacter* species are the main causative agents of infections in a majority of US hospitals. To combat all these continuing developments, a search for new and novel drugs scaffolds remains the high priority activity.

Eighty five years after the discovery of Penicillin in 1929, scientists all over the world continue to investigate natural products. The novelty of structures and scaffolds, their varied bioactivities plus their abilities to act as lead molecules is immense. According to Newman and Cragg (2012), in the years 1981–2010, ~50% of all small molecules originated from natural products. Mainly antibacterial, anticancer, antiviral and antifungals compounds from natural sources such as plant, fungi and bacteria themselves. The extraordinary advantages of natural products as sources of biotherapeutics is beyond question.

Though diverse chemical compounds with equally diverse scaffolds and bioactivities have been reported from fungi over the years, the vast group still remains to be fully exploited. Out of ~1 million different fungal species only ~100,000 have been described (Hawksworth and Rossman, 1997). Dreyfuss and Chapela (1994) estimated that endophytic fungi, alone could be ~1 million. The genetic diversity of fungal endophytes may be a major factor in the discovery of novel bioactive compounds (Gunatilaka, 2006). The true potential of these endophytes is yet to be trapped.

From the first reports of isolation from the *Lolium temulentum* typically known as Darnel (ryegrass) by Freeman (1904), to the latest one from Antarctic moss (Melo et al., 2014), endophytic fungi have attracted the attention of botanists, chemists, ecologists, mycologists, plant pathologists and pharmacologists. It is estimated that each and every of the almost 300,000 plants that exist, hosts one or more endophyte (Strobel and Daisy, 2003). They occur everywhere, from the Arctic to Antarctic and temperate to the tropical climates. Endophytes reside in internal tissues of living plants but this association does not cause any immediate, overt, negative effects on the host plant (Bacon and White, 2000). According to Aly et al. (2011), the endophyte-plant host relationship is a balanced symbiotic continuum, ranging from mutualism through commensalism to parasitism. Many endophytic fungi remain quiescent within their hosts until it stressed or begins to undergo senescence. At this juncture the fungi may turn pathogenic (Rodriguez and Redman, 2008).

The impact of endophytes on our lives is seen in several of ways; from an insecticidal bio fumigant from the *Muscodor albus*, against adults and larvae of potato tuber moths (Lacey and Neven, 2006) to synthesis of “myco-diesel” by *Gliocladium roseum*, in the hope of alternate fuel options (Strobel et al., 2008). Between these extremes, endophytes has been shown to produce several pharmacologically important compounds such as antimycotics Cryptocin (Li et al., 2000) and Ambuic acid (Li et al., 2001), anticancer Torreyanic acid (Lee et al., 1996), Taxol (Strobel et al., 1996), anti-inflammatory Ergoflavin (Deshmukh et al., 2009), antidiabetic (nonpeptidal compound L-783,281) (Zhang et al., 1999), antioxidant Pestacin (Harper et al., 2003), Isopestacin (Strobel et al., 2002), antiviral Cytonic acids A and B (Guo et al., 2000), alkaloids and polyketides Sclerotinin A (Lai et al., 2013), Cryptosporioptide (Saleem et al., 2013), enzyme inhibitors- Fusaric acid derivatives (Chen et al., 2013), Anthraquinones (Hawas et al., 2012) and immunosuppressive agents Subglutinols A and B (Lee et al., 1995).

The need for novel antibacterials to combat this increasing variety of infections becomes a priority endeavor. Endophytic fungi may be an important source for such biotherapeutics like new antibacterials against *Mycobacterium tuberculosis* especially in poverty ridden tropical countries of Asia. Here the need could also involve a nutritional efforts to boost the immunity in the population. Many of the compounds with their host plants are shown in Table 1.

**Table 1:** **Antibacterial compounds reported from endophytic fungi**.

**Sr. No**.	**Fungus**	**Plant source**	**Compounds isolated**	Biological activity^*^	**References**
1	*Pestalotiopsis* sp.	Lichen *Clavaroids* sp	Ambuic acid **(1)**	Compound **(1)**, *S. aureus* (ATCC 6538) (IC_50_ 43.9 μM)	Ding et al., 2009
	*Pestalotiopsis* sp.	Lichen *Clavaroids* sp	Ambuic acid derivative **(2)**	Compound **(2)**, *S. aureus* (ATCC 6538) (IC_50_ 27.8 μM)	Ding et al., 2009
2	*Pestalotiopsis* sp.	*Rhizophora mucronata*	Pestalotiopsone A **(3)**	Compound **(3)**, *E. faecalis* (MIC 125–250 μg/mL)	Hemberger et al., 2013
3	*Pestalotiopsis mangiferae1*	*Mangifera indica*	4-(2,4,7-trioxa-bicyclo[4.1.0]heptan-3-yl) phenol **(4)**	Compound **(4)**, *B. subtilis* and *K. pneumoniae* (MIC 0.039 μg/mL), *E. coli* and *M. luteus* (MIC 1.25 μg/mL), *P. aeruginosa* (MIC 5.0 μg/mL).	Subban et al., 2013
4	*Pestalotia* sp. /Unicellular marine bacterium strain CNJ-328	Co-cultured endophytic algal marine fungus/Unicellular marine bacterium strain CNJ-328	Pestalone **(5)**	Compound **(5)**, MRSA (MIC 37 ng/mL), VRE (MIC 78 ng/mL)	Cueto et al., 2001
				Compound **(5)**, *S. aureus* strain SG511 (MIC 3.1 μg/mL), MRSA LT-1334 (MIC 6.25 μg/mL), *B.subtilis* 168 (MIC 1.6 μg/mL)	Augner et al., 2013
5	*Phomopsis longicolla*	*Dicerandra frutescens*	Dicerandrols A **(6)**, B **(7)**, and C **(8)**	Compounds **(6)**, **(7)** and **(8)**, *B. subtilis* (zones of inhibition of 11 mm, 9.5 mm and 8.0 mm respectively) *S. aureus* (zones of inhibition of 10.8, 9.5 and 7.0 mm respectively) at 300 μg/disk	Wagenaar and Clardy, 2001
6	*Phomopsis longicolla* strain C81	*Bostrychia radicans*	Dicerandrol C **(8)**	Compound **(8)**, *S.aureus* (ATCC 6538) and *S. saprophyticus* (ATCC 15305), (MIC of 1 and 2 μgmL)	Erbert et al., 2012
7	*Phomopsis longicolla* S1B4	Unidentified plant	Dicerandrol A **(6)**, Dicerandrol B **(7)**, Dicerandrol C **(8)**, Deacetylphomoxanthone B **(9)** Fusaristatin A **(10)**	Compounds **(6)**,**(7)**, **(8)**, **(9)** and **(10)**, *X. oryzae* KACC 10331 (MIC of 8, 16, >16, 4 and 128 μg/mL respectively) Compound **(6)**, *S. aureus* KCTC 1916, *B. subtilis* KCTC 1021, *Clavibacter michiganesis* KACC 20122, *Erwinia amylovora* KACC 10060, (MIC value of 0.25, 0.125, 1.0, and 32.0 μg/mL respectively)	Lim et al., 2010
8	*Phomopsis longicolla* S1B4	Unidentified plant	Dicerandrol A **(6)**, Dicerandrol B **(7)**, Dicerandrol C **(8)**, Deacetylphomoxanthone B **(9)** and Monodeacetylphomoxanthone B **(11)**	Compound **(11)**, *X. oryzae* (MIC of 32 μg/mL)	Choi et al., 2013
9	*Phomopsis* sp. BCC 1323	Unidentified plant	Phomoxanthone A **(12)** and B **(13)**,	Compounds **(12)** and **(13)**, *M. tuberculosis* H37Ra strain (MIC of 0.5 and 6.25 μg/mL respectively)	Isaka et al., 2001
10	*Phomopsis* sp.	*Costus* sp.	Phomoxanthone A **(12)**	Compound **(12)**, *B. megaterium* (Zone of inhibition of 3- 4 against the concentration of 10 mg/mL)	Elsaesser et al., 2005
11	*Phomopsis* sp. (internal strain no. 7233)	*Laurus azorica*	Cycloepoxylactone **(14)**, Cycloepoxytriol B **(15)**	Compounds **(14)** and **(15)**, *B. megaterium* (moderate activity)	Hussain et al., 2009a
12	*Phomopsis* sp.	*Teucrium scorodonia*	Phomosines A-C **(16–18)**	Compound **(16–18)**, *B. megaterium* and *E. coli* (moderate activity *in vitro* using 6 mm filter paper disc with 50 μl of a 15 mg/mL solution)	Krohn et al., 1995
13	*Phomopsis* sp.	*Ligustrum vulgare*	Phomosines A-C **(16–18)**	Compounds **(16–18)**, *B. megaterium* (zone of inhibition with 10, 10 and 7 mm using 6 mm filter paper disc with 50 μg of compound).	Krohn et al., 2011
14	*Phomopsis* sp.	*Adenocarpus foliolosus*	Phomosine A **(16)** and Phomosine G **(19)**	Compounds **(16)** and **(19)**, *B. megaterium* (moderate antibacterial activity)	Dai et al., 2005
15	*Phomopsis* sp.	*Notobasis syriaca*	Phomosine K **(20)** 2-hydroxymethyl-4β ,5α,6β -trihydroxycyclohex-2-en **(21)**, (−)-Phyllostine **(22)**, (+)-Epiepoxydon **(23)**, and (+)-Epoxydon monoacetate **(24)**	Compound **(20)**, *Legionella pneumophila* Corby, *E. coli* K12 and *B. megaterium* (strong activity). Compound **(21)**,**(22)**,**(23)** and **(24)**, *E. coli* K12 and *B. megaterium* (moderately active)	Hussain et al., 2011
16	*Phomopsis* sp.	*Santolina chamaecyparissus*	Phomopsinone B **(25)** and C **(26)**	Compounds **(25)** and **(26)**, *E.coli*, and *B. megaterium* (moderately active)	Hussain et al., 2012b
17	*Phomopsis* sp	Unidentified plant	Phomochromone A **(27)**, B **(28)**, Phomotenone **(29)**, (1S,2S,4S)-trihydroxy-p-menthane **(30)**	Compounds **(27–30)**, *E. coli* and *B. megaterium* (active)	Ahmed et al., 2011
18	*Phomopsis* sp.	*Cistus salvifolius*	Pyrenocines J-M **(31–34)**	Compounds **(31–34)**, *E. coli* and *B. megaterium* (active)	Hussain et al., 2012a
19	Endophytic fungi	*Urobotrya siamensis, Grewia* sp., *Mesua ferrea, Rhododendron lyi, Tadehagi* sp., and *Gmelina elliptica*	3-Nitropropionic acid **(35)**	Compound **(35)**, *Mycobacterium tuberculosis* H37Ra (MIC of 3.3 μM)	Chomcheon et al., 2005
20	Phomopsis sp.	*Erythrina crista-galli*	Phomol **(36)**	Compound **(36)**, *A. citreus* and *C. insidiosum* (MIC of 20 and 10 μg/mL respectively)	Weber et al., 2004
21	*Phoma* sp.	*Saurauia scaberrinae*	Phomodione **(37)**	Compound **(37)**, *S. aureus* (MIC of 1.6 μg/mL)	Hoffman et al., 2008
22	*Phoma* sp.	*Salsola oppostifolia*	Epoxydines B **(38)**, Epoxydon **(39)**, (4R,5R,6S)-6-acetoxy-4,5-dihydroxy-2-(hydroxymethyl) cyclohex-2-en-1-one **(40)**, 2-chloro-6-(hydroxymethyl) benzene-1,4-diol **(41)**, antibiotic ES-242-1 **(42)**	Compounds **(38–42)**, *E. coli* and *B. megaterium* (active)	Qin et al., 2010
23	Phoma sp.	*Salsola oppositifolia*	(+)-Flavipucine **(43)**, (−)-Flavipucine **(44)**	Compound **(43)**, *B. subtilis, S. aureus, E. coli* (zone of inhibition of 16, 17, and 11 mm, respectively in disc diffusion assay at 15 μg/6 mm disc), Compound **(44)**, *B. subtilis* and *E. coli* (MICs of 25 μg/ mL)	Loesgen et al., 2011
24	Phoma sp. NRRL 46751,	*Saurauia scaberrinae*	Phomapyrrolidone B **(45)**, C **(46)**	Compound **(45)** and **(46)**, *M. tuberculosis* H37Pv Microplate Alamar Blue assay (MABA) for replicating cultures (with MIC of 5.9 and 5.2 μg/mL respectively) Low oxygen recovery assay (LORA) (MIC 15.4 and 13.4 μg/mL respectively for nonreplicating)	Wijeratne et al., 2013
25	*Colletotrichum gloeosporioides*	*Artemisia mongolica*	Colletotric acid **(47)**	Compound **(47)**, *B. subtilis, S. aureus*, and *S. lutea* (MIC of 25, 50, and 50 μg/mL)	Zou et al., 2000
26	*Colletotrichum* sp.	*Ilex canariensis*	(22E,24R)-19(10–>6)-abeo-ergosta-5,7,9,22-tetraen-3beta-ol **(48)**, (22E,24R)-ergosta-4,7,22-trien-3-one **(49)**, (22E,24R)-ergosta-4,6,8(14),22-tetraen-3-one **(50)**, (22E,24R)-ergosta-7,22-dien-3beta,5alpha,6beta-triol **(51)**, (22E,24R)-6-acetoxy-ergosta-7,22-dien-3beta,5alpha,6beta-triol **(52)**, and (22E,24R)-3,6-diacetoxy-ergosta-7,22-dien-3beta,5alpha,6beta-triol **(53)**	Compounds **(48–53)**, *E. coli* and *B. megaterium* (active at the concentration of 0.05 μg/filter paper disc of 6 mm diameter)	Zhang et al., 2009
27	*Coniothyrium* sp.	*Sideritis chamaedryfolia*	1-hydroxy-5-methoxynaphthalene **(54)**, 1,5-dimethoxy-4-nitronaphthalene **(55)**, 1- hydroxy-5-methoxy-2,4-dinitronaphthalene **(56)**	Compounds **(54–56)**, *E. coli* and *B. megaterium* (active)	Krohn et al., 2008a
28	*Coniothyrium cereale*	Marine green alga *Enteromorpha* sp.	(−)-Trypethelone **(57)**	Compound **(57)** *Mycobacterium phlei, S. aureus*, and *E. coli*, (at 20 μg/disk zones of inhibition of 18, 14, and 12 mm respectively)	Elsebai et al., 2011
29	*Coniothyrium* sp	*Salsola oppostifolia*	Pachybasin **(58)**, 1,7-Dihydroxy-3-methyl-9,10-anthraquinone **(59)**, Phomarin **(60)** 1-Hydroxy-3-hydroxymethyl-9,10-anthraquinone **(61)** and Coniothyrinones A-D **(62- 65)**	Compounds **(58–65)**, *E. coli* and *B. megaterium* (active at 50 μg/9 mm filter paper disc dissolved in acetone)	Sun et al., 2013a
30	*Diaporthe phaseolorum*	*Laguncularia racemosa*	3-Hydroxypropionic acid **(66)**	Compound **(66)**, *S. aureus* and *S. typhi* (MIC of 64 μg/ mL)	Sebastianes et al., 2012
31	*Botryosphaeria mamane* PSU-M76	*Garcinia mangostana*	Botryomaman **(67)**, 2,4-Dimethoxy-6-pentylphenol **(68)**,(*R)*- (−)-Mellein **(69)**, Primin **(70)**, *cis*-4-hydroxymellein **(71)**, *trans*-4-hydroxymellein **(72)**, and 4,5-dihydroxy-2-hexenoic acid **(73)**	Compounds **(67–73)***, S. aureus* ATCC 25923 and MRSA SK1 (active). Compound (70) *S. aureus* ATCC 25923 and MRSA SK1 (MIC values of 8 μg/mL)	Pongcharoen et al., 2007
32	*Microdiplodia* sp	*Lycium intricatum*	Diversonol **(74)**, Microdiplodiasol **(75)**, Microdiplodiasone **(76)**, Microdiplodiasolol **(77)** (−)-Gynuraone **(78)** and Ergosterol **(79)**	Compounds **(74–79)**, *Legionella pneumophila* (active)	Siddiqui et al., 2011
33	*Microdiplodia* sp. KS 75-1	*Pinus* sp.	7,8-dihydonivefuranone A **(80)**, 6(7)-dehydro-8-hydroxyterrefuranone **(81)**, 6-hydroxyterrefuranone **(82)**, Nivefuranones A **(83)**	Compounds **(80–83)**, *S. aureus* NBRC 13276 (zone of inhibition of 15, 15, 16 and 15 mm respectively at 40 μg/per disc of 8 mm diameter)	Shiono et al., 2012
34	*Microsphaeropsis arundinis*	*Pinus* sp.	1β -hydroxy-α-cyperone **(84)**	Compound **(84)**, *S. aureus* (CGMCC1.2465) (MIC 11.4 μg/mL)	Luo et al., 2013
35	*Microsphaeropsis* sp. (strain 8875)	*Lycium intricatum*	Microsphaeropsone A **(85)**, Microsphaeropsone C **(86)**, Citreorosein **(87)**	Compounds **(85–87)**, *E.coli* and *B. megaterium* (active)	Krohn et al., 2009
36	*Microsphaeropsis* sp. (internal strain no. 7177)	*Zygophyllum fortanesii*	Fusidienol A **(88)**, aromatic xanthones **(89)**, 3,4-dihydroglobosuxanthone A **(90)**	Compounds **(88–90)**, *E.coli* and *B. megaterium* (active)	Krohn et al., 2009
37	*Dinemasporium strigosum*	*Calystegia sepium*	Dinemasones A **(91)**, B **(92)**	Compounds **(91)** and **(92)**, *B. megaterium* (active)	Krohn et al., 2008b
38	*Cytospora* sp. CR200 and *Diaporthe* sp. CR146	*Conocarpus erecta* and *Forsteronia spicata*	Cytosporones D **(93)**, E **(94)**	Compound **(93)**, *S. aureus, E. faecalis* and *E. coli* (MIC of 8, 8, and 64 μg/mL respectively) Compound **(94)**, *S. aureus, E. faecalis* and *E. coli* (MIC of 8, 8, and 64 μg/mL respectively)	Brady et al., 2000
39	*Cytospora* sp. CR200	*Conocarpus erecta*	Cytosporones D **(93)**, E **(94)** Cytoskyrin A **(95)**	Compound A **(95)**, *S. aureus* ATCC 29923, *S. aureus* ATCC6538P, *S. aureus* #310 (MRSA), *E. faecium* #379 (VREF), *E. faecium* #436 (VSEF), *B. subtilis* BGGS1A1, *E. coli imp* BAS849, *E. coli* BAS849, *E. coli* ATCC25922, *K. pneumoniae* ATCC 10031, *P. aeruginosa* ATCC 27079 (MICs 0.03–0.25 μg/mL) and compound **(93)** and **(94)**, Above mentioned bacteria (MICs 8–64 μg/mL)	Singh et al., 2007
40	*Cytospora* sp.	*Ilex canariensis*	(R)-5-((S)-hydroxy(phenyl)-methyl) dihydrofuran-2(3H)-one **(96)** and its 6-acetate **(97)**, a new naphthalenone derivative **(98)**, (S)-5-((S)-hydroxy(phenyl)-methyl)dihydrofuran-2(3H)-one **(99)**, (S)-5-benzyl-dihydrofuran-2(3H)-one **(100)**, 5-phenyl-4-oxopentanoic acid **(101)**, gamma-oxo-benzenepentanoic acid methyl ester **(102)**, 3-(2,5-dihydro-4-hydroxy-5-oxo-3-phenyl-2-furyl)propionic acid **(103)**, (3R)-5-methylmellein **(104)**, Integracins A **(105)** and B **(106)**	Compounds **(96–106)**, *B. megaterium* (zone size in the range of 15–25 mm when 50 μl of the solution (0.05 mg substance) were pipetted onto a sterile filter disc 9 mm)	Lu et al., 2011
41	*Chaetomium globosum*	*Viguiera robusta*	Chaetoglobosin B **(107)**	Compound **(107)**, *S. aureus* (MIC 120 μg/mL) and *E. coli* (MIC 189 μg/mL)	Momesso et al., 2008
42	*Chaetomium globosum* strain IFB-E036	*Cynodon dactylon*	Chaetoglocins A **(108)**, B **(109)**	Compounds **(108)** and **(109)**, *B. subtilis, S. pyogens, M. luteus* and *M. smegmatis* (MIC between 8 and 32 μg/mL)	Ge et al., 2011
43	*Chaetomium globosum* SNB-GTC2114	*Paspalum virgatum*	Acremonisol A **(110)**, Semicochliodinol A **(111)**, Cochliodinol **(112)**	Compounds **(110)**, **(111)** and **(112)**, *S. aureus* ATCC 29213 (MIC of 64, 2 and 4 μg/mL respectively)	Casella et al., 2013
44	*Lewia infectoria* SNB-GTC2402	*Besleria insolita*	Pyrrocidine A **(113)** Pyrrocidine B **(114)** Pyrrocidine C **(115)**, Alterperylenol **(116)**	Compounds **(113–114)**, *S. aureus* ATCC 29213, (MIC value of 5 μg/ mL) Compounds **(115)**, and **(116)**, *S. aureus* ATCC 29213 (MIC of 2, and 32 μg/mL respectively)	Casella et al., 2013
45	*Xylaria* sp.	*Ginkgo biloba*	7-amino-4-methylcoumarin **(117)**	Compound **(117)**, *S. aureus, E. coli, S. typhi, S. typhimurium, S.enteritidis, A. hydrophila, Yersinia* sp., *V. anguillarum, Shigella* sp., and *V. parahaemolyticus* (MIC of 16, 10, 20 15, 8.5, 4, 12.5, 25, 6.3, and 12.5 μg/mL respectively)	Liu et al., 2008
46	*Xylaria* sp.	*Torreya jackii*	1-(xylarenone A) xylariate A **(118)**, Xylarioic acid B **(119)**, Xylariolide A **(120)**, Xylariolide B **(121)**, Xylariolide C **(122)**, Me xylariate C **(123)**, xylariolide D **(124)**, Taiwapyrone **(125)**	Compounds **(118–125)**, *E. coli* ATCC 25922, *B. subtilis* ATCC 9372, and *S. aureus* ATCC 25923 (MIC values above 10 μg/mL)	Hu et al., 2010
47	*Cryptosporiopsis* sp.,	*Viburnum tinus*	Cryptosporioptide **(126)**	Compound **(126)**, *B.megaterium* (with a 9 mm radius of the zone of inhibition (50 μg/9 mm paper disc)	Saleem et al., 2013
48	*Microdochium bolleyi*	*Fagonia cretica*	Monocerin **(127)**, (12*S)*-12-Hydroxymonocerin **(128)**, Isocoumurin **(129)**	Compounds **(127–129)**, *E. coli* and *B. megaterium* (active)	Zhang et al., 2008a
49	*Chalara* sp. strain 6661	*Artemisia vulgaris*	Isofusidienol A, B, C, and D **(130–133)**	Compounds **(130)** and **(131)**, *B. subtilis* (with zone of inhibition 23 and 22 mm respectively, (15 μg/6-mm filter disks). Compound **(130)**, *S. aureus* and *E. coli* (With zone of inhibition 9 and 8 mm, (15 μg per 6-mm filter disks). Compounds **(132)** and **(133)**, *B. subtilis* (With zone of inhibition of 9 and 8 mm against (15 μg per 6-mm filter disks)	Loesgen et al., 2008
50	*Blennoria* sp.	*Carpobrotus edulis*	Secalonic acid B **(134)** Blennolides A **(135)**, and B **(136)**	Compounds **(134–136)**, *B. megaterium* (active), Compounds **(135)** and **(136)**, *E.coli* (active)	Zhang et al., 2008b
51	*Preussia* sp.	*Aquilaria sinensis*	Spiropreussione A **(137)**	Compond **(137)**, *S. aureus* (CMCC B26003) (Zone of inhibition of 16.4 ± 0.3 mm (*n* = 3) at 5 μg/disk, MIC 25 μM)	Chen et al., 2009
52	*Guignardia* sp. IFB-E028	*Hopea hainanensis*	Monomethylsulochrin **(138)**, Rhizoctonic acid **(139)**, Guignasulfide **(140)**	Compounds **(138–140)**, *Helicobacter pylori* (MIC values of 28.9, 60.2, and 42.9 μM, respectively)	Wang et al., 2010
53	*Pichia guilliermondii* Ppf9	*Paris polyphylla* var. *yunnanensis*	Helvolic acid **(141)**	Compound **(141)**, *A. tumefaciens, E. coli, P. lachrymans, R. solanacearum, X. vesicatoria, B. subtilis, S. aureus* and *S.haemolyticus*,(MICs 1.56, 3.13, 3.13, 1.56, 1.56,3.13, 50, and 6.25 μg/mL)	Zhao et al., 2010
54	*Sordariomycete* sp.strain B5	*Eucommia ulmoides* Oliver	Chlorogenic acid **(142)**	Compound **(142)**, (Antibacterial, antifungal, antioxidant, and antitumor activities)	Chen et al., 2010
55	*Biscogniauxia formosana* BCRC 33718	*Cinnamomum* sp.	Biscogniazaphilones A **(143)** and B **(144)**, N-trans-feruloy-3-O-methyldopamine **(145)** 5-hydroxy-3,7,40-trimethoxyflavone **(146)** 4-methoxycinnamaldehyde **(147)** methyl 3,4-methylenedioxycinnamate **(148)** 4-methoxy-trans-cinnamic acid **(149)**	Compounds **(143)** and **(144)**, *M. tuberculosis* strain H37Rv. (MIC values of ≤5.12 and ≤2.52 μg/mL). Compounds **(145–149)**, *M. tuberculosis* strain H37Rv. (MICs 12.5, 25.0, 42.1, 58.2, and 50.0 μg/mL)	Cheng et al., 2012
56	Dothideomycete sp.	*Tiliacora triandra*	Dothideomycetide A **(150)**	Compound **(150)**, *S. aureus* ATCC 25923 and MRSA ATCC 33591 (MICs 128 and 256 μg/mL respectively)	Senadeera et al., 2012
57	*Eurotium cristatum EN-220*	*Sargassum thunbergii*	Cristatumins A **(151)**, Tardioxopiperazine A **(152)**	Compounds **(151)** and **(152)**, *E. coli* and *S. aureus* (MICs 64 and 8 μg/mL)	Du et al., 2012
58	*Aspergillus* sp. CY725	*Cynodon dactylon*	Helvolic acid **(141)**, Monomethylsulochrin **(138)**, ergosterol **(79)**, 3β -hydroxy-5α,8α-epidioxy- ergosta-6,22-diene **(153)**	Compounds **(141)**, **(138)**, **(79)**, and **(153)**, *H. pylori* (MICs of 8.0, 10.0, 20.0, and 30.0μg/mL respectively). Compound **(141)** *Sarcina lutea* and *S. aureus* (MICs 15.0 and 20.0 μg/mL respectively)	Li et al., 2005
59	*Aspergillus* sp.	Mixed cultured mycelia of two marine-derived mangrove epiphytic fungi	Aspergicin **(154)**, Neoaspergillic acid **(155)**	Aspergicin **(154)**, *S. aureus, S. epidermidis, B. subtilis, B. dysenteriae, B. proteus*, and *E. coli*, (MIC of 62.5, 31.25 15.62, 15.62 62.5, and 31.25 μg/mL respectively) Compound **(155)**, *S. aureus, S. epidermidis, B. subtilis, B. dysenteriae, B. proteus*, and *E. coli*, (MIC of 0.98, 0.49, 1.95, 7.8, 7.8, and 15.62 μg/mL respectively)	Zhu et al., 2011
60	*Aspergillus* sp.	*Bruguiera gymnorrhiza*	Aspergillumarin A **(156)**, B **(157)**	Compounds **(156)** and B **(157)**, *S. aureus* and *B. subtilis* (active at 50 μg/mL)	Li et al., 2012
61	*Aspergillus versicolor*	Brown alga *Sargassum thunbergii*	Brevianamide M **(158)**, 6,8-di-O-methylaverufin **(159)**, 6-O-methylaverufin **(160)**,	Compounds **(158–160)**, *S. aureus* and *E. coli* (active)	Miao et al., 2012
62	*Aspergillus versicolor*	Red Sea green alga *Halimeda opuntia*	Isorhodoptilometrin-1-Me ether **(161)**, Siderin **(162)**,	Compounds **(161, 162)***, B. cereus, B. subtilis* and *S. aureus* (actve at 50 μg/disc of 9 mm)	Hawas et al., 2012
63	*Aspergillus wentii* pt-1	Red alga *Gymnogongrus flabelliformis*	Yicathin B**(163)**, Yicathin C **(164)**	Compound **(163)**, *E. coli* (inhibition diameter 9 mm) and **(164)**, *E. coli* (12.0 mm) and *S. aureus* (7.5 mm) at 10 mg/disk	Sun et al., 2013b
64	*Aspergillus* sp. EJC08	*Bauhinia guianensis*	Fumigaclavine C **(165)**, Pseurotin A **(166)**	Compound **(165)**, *B. subtilis, E. coli, P. aeruginosa* and *S. aureus* (MICs 7.81 62.50 31.25 15.62 μg/mL). Compound **(166)**, *B. subtilis, E. coli, P. aeruginosa* and *S. aureus* (MICs 15.62 31.25 31.25 15.62 μg/mL)	Pinheiro et al., 2013
65	*Penicillium sclerotiorum PSU-A13*.		(+)-Sclerotiorin **(167)**,	Compound **(167)**, *S. aureus* sub sp.*aureus* ATCC 29213, (MIC 128 μg/mL)	Lucas et al., 2007; Arunpanichlert et al., 2010
66	*Penicillium janczewskii*	*Prumnopitys analina*	Pseurotin A **(166)**	Compond **(166)**, *E. carotovora* and *P. syringae*, (IC_50_ values of 220 and 112 μg/mL)	Schmeda-Hirschmann et al., 2008
67	*Penicillium citrinum*strain ZD6	*Bruguiera gymnorrhiza*	Emodin **(168)**, Erythritol **(169)**	Compounds **(168)** and **(169)**, *B. subtilis* (MICs 25μg/mL and 50μg/mL respectively), Compound **(168)** *P. aeruginosa* (MIC 100 μg/mL)	Li et al., 2010
68	*Penicillium chrysogenum* QEN-24S	Marine red alga *Laurenciasp*	Conidiogenone B **(170)**, Conidiogenol **(171)**	Compound **(170)**, MRSA, *P. fluorescens*, P. *aeruginosa*, and *S. epidermidis* (each with a MIC value of 8 μg/mL), Compound **(171)**, *P. fluorescens* and *S. epidermidis* (each with a MIC value of 16 μg/mL)	Gao et al., 2011
69	*Penicillium chrysogenum* MTCC 5108	*Porteresia coarctata*	3,1'-didehydro-3[2”(3'”,3'”-dimethyl-prop-2-enyl)-3”-indolylmethylene]-6-Me pipera-zine-2,5-dione **(172)**	Compound **(172)**, *Vibrio cholera* MCM B-322 (active)	Devi et al., 2012
70	*Penicillium citrinum*	*Ocimum tenuiflorum*	Perinadine A **(173)**, Alternariol **(174)**, Citrinin **(175)**	Compounds **(173–175)**, *S. aureus* ATCC 29213 (MIC 64 μg/mL)	Lai et al., 2013
71	*Fusarium* sp. IFB-121	*Quercus variabilis*	(2S,2'R,3R,3'E,4E,8E,10E)-1-O-β -D-glucopyranosyl-2-N-(2'-hydroxy-3'-octadecenoyl)-3-hydroxy-9-methyl-4,8,10-sphingatrienine **(176)**, and (2S,2'R,3R,3'E,4E,8E)-1-O-β -D-glucopyranosyl-2-N-(2'-hydroxy-3'-octadecenoyl)-3-hydroxy-9-methyl-4,8-sphingadienine **(177)**	Compound **(176)** and **(177)**, *B. subtilis, E. coli* and *P. fluorescens* (MICs 3.9, 3.9, and 1.9 μg/mL and 7.8, 3.9, and 7.8 μg/mL respectively)	Shu et al., 2004
72	*Fusarium* sp. YG-45	*Maackia chinensis*	Fusapyridon A **(178)**	Compound **(178)**, *P. aeruginosa* and *S. aureus*, (MICs 6.25 and 50 μg/mL respectively)	Tsuchinari et al., 2007
73	*Fusarium redolens* Dzf2	*Dioscorea zingiberensis*	Beauvericin **(179)**	Compound **(179)***, B. subtilis, E. coli, S. hemolyticus, P. lachrymans, A. tumefaciens*, and *X. vesicatoria* (IC_50_ values between 18.4 and 70.7 μg/mL)	Xu et al., 2010b
74	*Fusarium oxysporum*	*Cinnamomum kanehirae*	Beauvercin**(179)**, (−)-4, 6'-anhydrooxysporidinone **(180)**	Compound **(179)**, MRSA and *B.subtilis* (MICs 3.125 μg/mL). Compound **(180)**, MRSA (MIC 100 μg/mL) and *B.subtilis* (MIC 25 μg/mL)	Wang et al., 2011
75	*Fusarium* sp. BCC14842	Bamboo	Javanicin **(181)**, 3-O-methylfusarubin **(182)**, compounds **(183)** and **(184)**	Compound **(181)**, **(183)**, antimycobacterial activity (MICs of 25μg/mL) Compound **(182)** and **(184)**, antimycobacterial activity (MICs of 50 μg/mL)	Kornsakulkarn et al., 2011
76	*Fusarium* sp.	Mangrove plant	Cadmium **(185)** and copper **(186)** metal complexes of Fusaric acid	Compounds **(185) (186)**, *Mycobacterium bovis* BCG (MICs 4μg/mL) and the *M. tuberculosis* H37Rv strain (MICs 10 μg/mL)	Pan et al., 2011
77	*Fusarium solani*	*Ficus carica*	Fumitremorgin B **(187)**, Fumitremorgin C **(188)**, Helvolic acid **(141)**, Bisdethiobis (methylthio) gliotoxin **(189)**, Bis-N-norgliovietin **(190)**, Gliotoxin **(191)**	Compounds **(187–191)**, *B. subtilis, S. aureus*, and *E. coli* and *P. aeruginosa* (MIC in the range of 0.5–16 μg/mL)	Zhang et al., 2012
78	Coculturing the fungal endophyte *Fusarium tricinctum* with the bacterium *Bacillus subtilis* 168 trpC2	*Aristolochia paucinervis*	Lateropyrone **(192)**, Enniatins B1 **(193)**, A1 **(194)**	Compounds **(193)** **(194)**, *B. subtilis* (MICs 16 and 8 μg/mL respectively), and *S. aureus, S. pneumoniae*, and *E. faecalis* (MICs in the range 2-8 μg/mL). Compound **(192)**, *B. subtilis, S. aureus, S. pneumoniae*, and *E. faecalis* (MICs in the range 2–8 μg/mL). Compounds **(192–194)**, were effective against a multiresistant clinical isolate of *S. aureus*	Ola et al., 2013
79	*Fusarium solani*	*Rheum palmatum* L.	Rhein **(195)**	Compound **(195)**, *S. aureus, S. aureus nor A, B. megaterium* 11561, *P. syringae* and *S. meliloti* (MICs in the range of 0.6–4 μg/mL)	You et al., 2013
80	*Fusarium proliferatum* BLH51	*Macleaya cordata*	Sanguinarine **(196)**	Sanguinarine **(196)**, 15 clinical isolates of *S. aureus* (MIC range of 3.12–6.25 μg/mL) Two reference strains ATCC 25923 (MIC are 3.12 μg/mL), ATCC 33591 (MIC 1.56 μg/mL)	Wang et al., 2014
81	*Trichoderma ovalisporum* strain PRE-5	*Panax notoginseng*	Shikimic acid **(197)**,	Compound **(197)** *S. aureus, Bacillus cereus, M. luteus* and *E. coli* (Active)	Dang et al., 2010
82	*Trichoderma* sp. PR-35	*Paeonia delavayi*	Trichoderic acid **(198)**, 2β -hydroxytrichoacorenol **(199)**, Cyclonerodiol **(200)**, Cyclonerodiol oxide **(201)**, and Sorbicillin **(202)**	Compounds **(198–202)**, *E. coli* and *S. albus* (MIA values in the range of 25– 150 mg/disk). Compounds **(198)**, **(200)**, and **(201)**, *S. sonnei* (MIA values in the range of 100- 150 μg/disk)	Wu et al., 2011
83	*Trichoderma asperellum*	*Panax notoginseng*	PF1022F **(203)**, Halobacillin **(204)**	Compounds **(203)** and **(204)**, *E.faecium* (CGMCC 1.2025) (IC_50_ 7.30 and 5.24 μM respectively), *S. aureus* COL (CGMCC 1.2465) (IC_50_ 19.02 and 14.00 μM respectively)	Ding et al., 2012
84	*Nigrospora* sp. MA75	*Pongamia pinnata*	Tetrahydrobostrycin **(205)**, 4-deoxytetrahydrobostrycin **(206)**, 3,6,8-trihydroxy-1-methylxanthone **(207)**, Griseophenone C **(208)** and 2,3-didehydro-19α-hydroxy-14-epicochlioquinone B **(209)**	Compound **(209)**, MRSA, *E. coli, P.aeruginosa, P. fluorescens*, and *S. epidermidis* (MICs 8, 4, 4, 0.5, and 0.5 μg/mL, respectively). Compound **(208)**, MRSA, *E. coli, P. aeruginosa*, and *P. fluorescens* (MICs 0.5, 2, 0.5, and 0.5 μg/mL respectively). Compound **(205)**, MRSA and *E. coli* (MIC 2 and 0.5 μg/mL, respectively), Compound **(206)**, *E. coli* (MIC 4 μg/mL) Compound **(207)**, *S. epidermidis* (MIC 0.5 μg/mL)	Shang et al., 2012
85	*Nigrospora* sp	Plant collected from South China Sea	4-deoxybostrycin **(210)**, Nigrosporin **(211)**	Compounds **(210)** and **(211)**, *M. tuberculosis* and clinical multidrug-resistant (MDR) *M. tuberculosis* strains (MICs <5 - > 60 μg/mL)	Wang et al., 2013b
86	*Periconia* sp.	*Taxus cuspidata*	Periconicins A **(212)** and B **(213)**	Compounds **(212)**, *B. subtilis, S. aureus, K. pneumoniae*, and *Salmonella typhimurium* (MICs in the range of 3.12–12.5 μg/mL) Compounds **(213)**, *B. subtilis, S. aureus, K. pneumoniae*, and *Salmonella typhimurium* (MICs in the range of 25–50 μg/mL)	Kim et al., 2004
87	*Periconia* sp.	*Piper longum*	Piperine **(214)**	Compound **(214)**, *M. tuberculosis* and *M. smegmetis* (MIC of 1.74 and 2.62 μg/mL respectively)	Verma et al., 2011
88	*Periconia siamensis* CMUGE015	*Thysanoleana latifolia*	Modiolide A, (5, 8-dihydroxy-10-methyl-5, 8, 9, 10-tetrahydro-2H-oxecin-2-one) **(215)**, 4-Chromanone, 6-hydroxy-2-methyl- (5CI) **(216)**	Compound **(215)**, *B. cereus, L. monocytogenes*, MRSA, *P. aeroginosa* and *E. coli* (MIC of 3.12, 6.25, 25.00, 12.50 and 50.00 μg/mL respectively) Compound **(216)**, *B. cereus, L. monocytogenes*, MRSA, *P. aeruginosa* and *E. coli* (MIC of 6.25, 12.50, 50.00 25.00, 12.50 and 100.00 μg/mL respectively)	Bhilabutra et al., 2007
89	*Alternaria* sp.	*Sonneratia alba*	Xanalteric acids I **(217)** and II **(218)**, Altenusin **(219)**	Xanalteric acids I **(217)** and II **(218)**, Multidrug-resistant *S. aureus* (MIC 250–125 μg/mL respectively). Altenusin **(219)**, MRSA, *S. pneumonia, E. faecium, E. cloacae and A. faecalis* (MIC values of 31.25–125 μg/mL)	Kjer et al., 2009
90	*Nodulisporium* sp.	*Juniperus cedre*	1-(2,6-dihydroxyphenyl)butan-1-one **(220)**	Compound **(220)**, *B. megaterium* (zone of inhibition 15 mm at a concentration of 0.25 mg/filter disc)	Dai et al., 2006
91	*Nodulisporium* sp.	*Erica arborea*	Nodulisporins D-F **(221–223)**, benzene- 1,2,3-triol **(224)**	Compounds **(221–224)**, *B. megaterium* (Active)	Dai et al., 2009b
92	*Acremonium zeae*	Maize plant	Pyrrocidine A **(113)**	Compound **(113)**, *C. michiganense* subsp. *nebraskense*, (MIC 1–2 μg/mL), *B. mojavensis* (MIC 1–2 μg/mL) and *P. fluorescens* (MIC 1–2 μg/mL)	Wicklow and Poling, 2009
93	*Rhizoctonia* sp. (Cy064),	*Cynodon dactylon*	Rhizoctonic acid **(139)**, Monomethylsulochrin **(138)**, Ergosterol **(79)**, 3β ,5α,6β -trihydroxyergosta-7,22-diene **(225)**	Compounds **(139, 138, 79, 225)**, Five clinical and one reference isolate of *H. pylori* (ATCC 43504) (MICs in the range of 10.0–30.0 μg/mL)	Ma et al., 2004
94	*Ulocladium* sp.	Lichens	Ophiobolins P **(226)**, T **(227)**	Ophiobolins P **(226)**, *B. subtilis* and MRSA (MIC of 62.5 and 31.3 μg/mL respectively). Ophiobolin T **(227)**, *B. subtilis* and MRSA *S. aureus* and Bacille Calmette-Guerin strain (MIC of 31.3 15.6 and 31.3 μg/mL respectively)	Wang et al., 2013a
95	*Chloridium* sp	*Azadirachta indica*	Javanicin **(181)**	Javanicin **(181)**, *P. fluorescens* and *P. aerugenosa* (MIC of 2 μg/mL)	Khrawar et al., 2009
96	*Botryosphaeria rhodina* PSU-M35 and PSU-M114	*Garcinia mangostana*	(3S)-lasiodiplodin **(228)**, (R)-(−)-mellein **(229)**, cis-(3R,4R)-(−)-4-hydroxymellein **(230)**, trans-(3R,4S)-(−)-4-hydroxymellein **(231)**, (R)-(−)-5-hydroxymellein **(232)**	Compound **(228)**, *S. aureus* and MRSA (MICs 64 and 128 μg/mL respectively), Compounds **(229–232)**, *S. aureus* and MRSA (MIC value of >128 μg/mL)	Rukachaisirikul et al., 2009
97	*Fusidium* sp.	leaves of *Mentha arvensis*	Fusidilactones D **(233)**, E **(234)**	Compounds **(233–234)**, *E. coli* and *B. megaterium* (active)	Qin et al., 2009
98	*Hyalodendriella* sp Ponipodef12	hybrid “Neva” of *Populus deltoides* Marsh × *P. nigra* L.	Palmariol B **(235)**, 4-hydroxymellein **(236)**, alternariol 9-methyl ether **(237)**, Botrallin **(238)**	Compounds **(235–238)***, A. tumefaciens* (IC_50_ values ranged from 18.22 μg/mL to 87.52 μg/mL). *B. subtilis, P. lachrymans, R. solanacearum, X. vesicatoria*, (MIC 19.22 μg/mL to 98.47 μg/mL, 16.18 μg/mL to 92.21 μg/mL, 16.24 μg/mL to 85.46 μg/mL, 17.81 μg/mL to 86.32 μg/mL, respectively)	Meng et al., 2012
99	*Stemphylium globuliferuman*	*Mentha pulegium (Lamiaceae)*	Alterporriol N **(239)**, alterporriol D **(240)**, alterporriol E **(241)**	Compound **(239)**, MRSA and *E. faecalis* (MIC of 62.5 and 15.63μg/mL). Compound **(240)**, MRSA and *S. pneumonia* (MIC of 31.25 μg/mL each) Compound **(241)**, *S. pneumonia, E. faecalis* and *E.cloacae* (MIC of 31.25 μg/mL each)	Debbab et al., 2009
100	Endophytic fungus, no. 1403	Mangrove	Bostrycin **(242)**	Bostrycin **(242)**, *B. subtilis* (Active)	Charudattan and Rao, 1982; Xu et al., 2010a
101	Costa Rican fungus CR115	*Daphnopsis americana*	Guanacastepene A **(243)**	Guanacastepene A **(243)**, MRSA and VRE (Active)	Singh et al., 2000
102	Costa Rican fungus CR115	*Daphnopsis americana*	Guanacastepene I **(244)**	Guanacastepene I **(244)**, *S. aureus* (Active in agar diffusion assay)	Brady et al., 2001
103	Endophytic fungus No. B77	Mangrove tree	Anhydrofusarubin **(245)**	Compound **(245)**, *S. aureus* (ATCC27154) (MIC 12.5 μg/mL)	Shao et al., 2008b
104	Endophytic fungus B77	seed of the mangrove sample Kandelia candel	3-O-methylfusarubin **(182)**, fusarubin **(246)**	Compounds **(182)** and **(246)**, *S. aureus* ATCC 27154 (MIC value of 50.0 and 12.5 μg/mL, respectively)	Shao et al., 2008a
105	Endophytic fungus PSU-N24	Garcinia nigrolineata	Compound 2 **(247)**, 9a –hydroxyhalorosellinia A **(248)** and desoxybostrycin **(249)**	Compound **(248)**, *M. tuberculosis* (MIC 12.50 μg/mL) Compounds **(247)** and **(249)**,(MIC 25 and 50 μg/mL, respectively)	Sommart et al., 2008
106	Endophytic fungus S20	*Cephalotaxus hainanensis* Li.	Indolyl-3-carboxylic acid **(250)**	Compound **(250)**, *S. aureus* and MRSA (Zones of inhibition 12 and 8 mm, respectively when 50 μl (10 mg/mL) of the compound was impregnated on sterile filter paper discs (6 mm diameter)	Dai et al., 2009a
107	Endophytic fungus S20	*Cephalotaxus hainanensis*.	5-acyl-2-methylpyrrole **(251)**	Compound **(251)**, *S. aureus* MRSA (Zone of inhibition of 12.0 mm and 10.0 mm respectively when 50 μl (10 mg/mL) of the compound was impregnated on sterile filter paper discs (6 mm diameter)	Dai et al., 2009c
108	Endophytic fungus Dzf12	*Dioscorea zingiberensis*	Diepoxin κ **(252)**, Diepoxin η **(253)**, Diepoxin ζ **(254)**	Compound **(252)**, *E. coli, A. tumefaciens, X. vesicatoria, P. lachrymans*and *B. subtilis* (MIC ranges from 50 to 100 μg/mL). The mixture of Commpounds **(253)** and **(254)**, *E. coli, A. tumefaciens, X. vesicatoria, P. lachrymans*and *B. subtilis* (MIC ranges from 5.0 to 12.5 μg/mL)	Cai et al., 2009
109	An unidentified Ascomycete	*Meliotus dentatus*	4-Hydroxyphthalide **(255)** 5-methoxy-7-hydroxyphthalide **(256)** (3R,4R)-cis-4-hydroxymellein **(257)**	Compounds **(255)** and **(256)**, *E. coli* (Active) and compounds **(256)** and **(257)**, *B. megaterium* (Active)	Hussain et al., 2009b
110	Unidentified ascomycete	*Arbutus unedo*	Pestalotheols E-H **(258–261)**, Anofinic acid **(262)**	Compounds **(258–262)**, *E. coli* and *B. megaterium* (Active)	Qin et al., 2011
111	Endophytic fungus A1	*Scyphiphora hydrophyllacea*	Guignardone I **(263)** and Guignardone B **(264)**	Guignardone I **(263)**, *S. aureus* (MRSA) and *S. aureus* (Zones of inhibition of 9.0 and 11.0 mm in diameter at 65 μM, respectively (the diameter of sterile filter paper discs was 6 mm). Guignardone B **(264)**, MRSA (zone of inhibition 8.0 mm against at 65 μM)	Mei et al., 2012
112	1223-D, an unclassified endophytic fungus	*Neomirandea angularis*	Mirandamycin **(265)**	Mirandamycin **(265)**, *E.coli* 25922, *P. aeruginosa* 27853, *K. pneumoniae* carbapenemase positive BAA-1705, MRSA BAA-976 and *V. cholerae* PW357 (MIC of 80, 80, > 80, 10 and 40 μg/mL respectively)	Ymele-Leki et al., 2012

* *Data as reported by authors*.

## Antibacterials from endophytic fungi

### Compounds from ascomycetes

**Figure 1:**
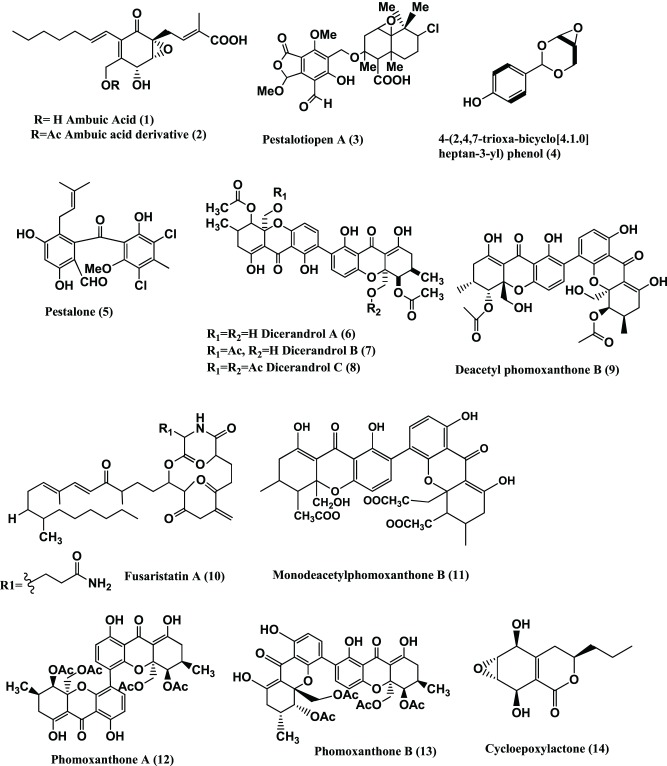
**Structures of antibacterial metabolites isolated from Ascomycetes (1–14)**.

Ascomycetes are an important class of fungi where there is formation of ascospores. Some genera of this class are prolific producer of bioactive metabolites. The genus *Pestalotiopsis* exists as an endophyte in most of the world's rainforests and is extremely biochemically diverse. Some examples of products from this group are Ambuic acid **(1)** and its derivative **(2)** (Figure 1) isolated from a *Pestalotiopsis* sp. of the lichen *Clavaroid* sp. Compounds **(1)** and **(2)** are active against *S. aureus* (ATCC 6538) with IC_50_ values of 43.9 and 27.8 μ M, respectively (the positive control Ampicillin showed an IC_50_ value of 1.40 μ M) (Ding et al., 2009).

Pestalotiopen A **(3)** (Figure 1), from *Pestalotiopsis* sp. of the Chinese mangrove *Rhizophora mucronata* exhibited moderate antimicrobial activity against *Enterococcus faecalis* with an MIC value between 125 and 250 μ g/mL (Hemberger et al., 2013).

A novel phenolic compound, 4-(2, 4, 7-trioxa-bicyclo [4.1.0] heptan-3-yl) phenol **(4)** (Figure 1) was isolated from *Pestalotiopsis mangiferae* associated with *Mangifera indica.* The compound exhibits activity against *Bacillus subtilis* and *K. pneumoniae* (MICs 0.039 μg/ml), *E. coli* and *Micrococcus luteus* (MICs 1.25 μg/ml) and *P. aeruginosa* (MIC 5.0 μg/ml). The positive control (Gentamycin) is showed activity against *B. subtilis*, *K. pneumoniae* and *M. luteus*, *E. coli*, and *P. aeruginosa* (MICs range 5.0–10.0 μg/ml). Transmission electron microscopy (TEM) analysis for mode of action of compound (**4**) showed that against the three human pathogens (*E. coli, P. aeruginosa*, and *K. pneumoniae*), morphological alterations took place: such as destruction of bacterial cells by cytoplasmic agglutination and formation of pores in cell wall membranes (Subban et al., 2013).

Pestalone **(5)** (Figure 1) is a chlorinated benzophenone antibiotic produced by a co-cultured *Pestalotia* sp./Unicellular marine bacterium strain CNJ-328. *Pestalotia* sp. was isolated from the brown alga *Rosenvingea* sp. collected in the Bahamas Islands. Pestalone exhibits potent activity against MRSA (MIC 37 ng/mL) and VRE (MIC 78 ng/mL), indicating that Pestalone should be evaluated in advanced models of infectious disease (Cueto et al., 2001). It is active against *S. aureus* strain SG511, MRSA LT-1334 and *Bacillus subtilis* 168 with MICs of 3.1, 6.25, and 1.6 μ g/mL respectively (Augner et al., 2013).

*Phomopsis*, another important genus exists as an endophyte in most plants and is also extremely biochemically diverse. Examples of bioactive metabolites from this endophyte are Dicerandrol A **(6)**, B **(7)**, and C **(8)** (Figure 1) from *Phomopsis longicolla* of the mint *Dicerandra frutescens*. They exhibit zones of inhibition of 11, 9.5, and 8.0 mm against B. *subtilis* respectively and 10.8, 9.5, and 7.0 mm respectively against *S. aureus* when tested at 300 μ g/disc (Wagenaar and Clardy, 2001).

Dicerandrol C **(8)** (Figure 1) was isolated from *Phomopsis longicolla* strain C81, from the red seaweed *Bostrychia radicans.* Dicerandrol C **(8)** had significant antimicrobial activity against *S. aureus* (ATCC 6538) and *Staphylococcus saprophyticus* (ATCC 15305), with MICs of 1 and 2 μ g /mL respectively (Erbert et al., 2012).

Dicerandrol A **(6)**, Dicerandrol B **(7)**, Dicerandrol C **(8)**, Deacetylphomoxanthone B (**9**) and Fusaristatin A **(10)** (Figure 1) were isolated from *Phomopsis longicolla* S1B4 from a plant sample from Hadong-gun, Kyungnam Province, South Korea. All of the above compounds show moderate to low antibacterial activities against *Xanthomonas oryzae* KACC 10331 with MICs of 8, 16, >16, 4, and 128 μ g/mL respectively. Dicerandrol A **(6)** is also active against *S. aureus* KCTC 1916, *B. subtilis* KCTC 1021, *Clavibacter michiganesis* KACC 20122, *Erwinia amylovora* KACC 10060, with MICs value of 0.25, 0.125, 1.0, and 32.0 μg/mL respectively (Lim et al., 2010). Monodeacetylphomoxanthone B (**11**) (Figure 1) was reported from the same culture along with compounds (**6–9**). It is active against *X. oryzae* with an MIC of 32 μ g/mL (Choi et al., 2013).

Phomoxanthones A (**12**) and B (**13)** (Figure 1) were obtained from *Phomopsis* sp. BCC 1323, of the leaf of *Tectona grandis* L., from the Mee Rim district of Chaingmai Province, Northern Thailand. These compounds show significant “*in vitro*” antitubercular activities with MICs of 0.5 and 6.25 μg/mL respectively against *Mycobacterium tuberculosis* H37Ra strain, in comparison to isoniazide and kanamycin sulfate (MICs of 0.050 and 2.5 μg/mL, respectively) that are used in clinics today (Isaka et al., 2001).

Phomoxanthone A (**12**) (Figure 1), was also isolated from a *Phomopsis* sp. of the stem of *Costus* sp. growing in the rain forest of Costa Rica. It has activity against *Bacillus megaterium* at a concentration of 10 mg/mL (radius of zone of inhibition of 3–4 cm) (Elsaesser et al., 2005).

**Figure 2:**
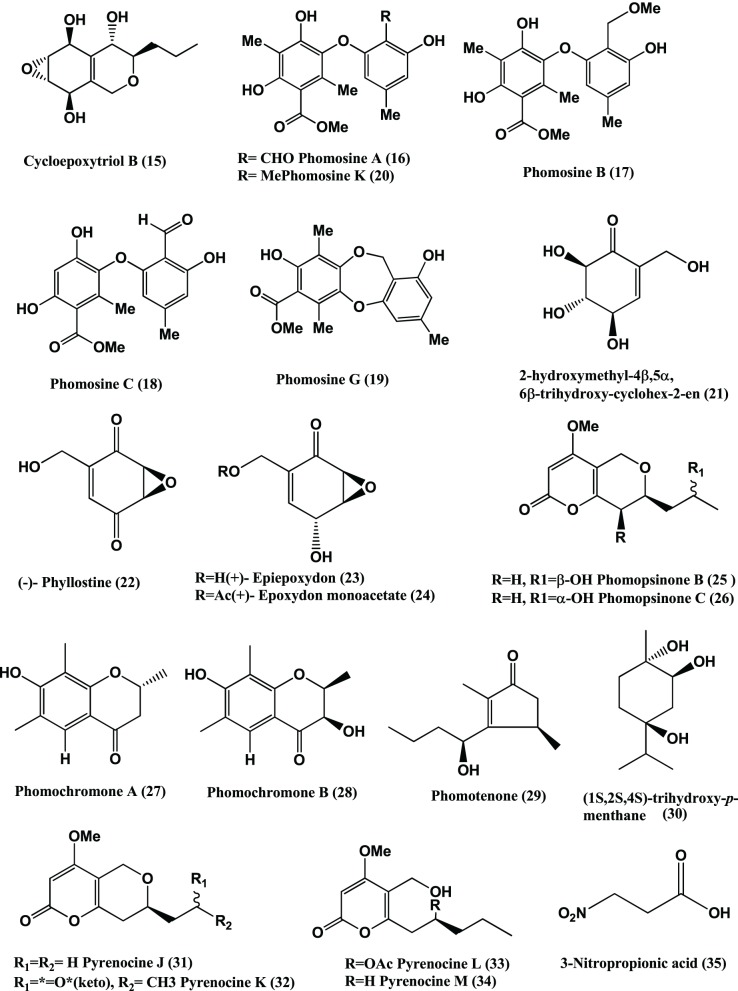
**Structures of antibacterial metabolites isolated from Ascomycetes (15–35)**.

Cycloepoxylactone (**14**) (Figure 1) and cycloepoxytriol B (**15**) (Figure 2) were detected from *Phomopsis* sp. (internal strain no. 7233) of *Laurus azorica*. They are moderately active against *B. megaterium* (Hussain et al., 2009a).

Phomosines A–C **(16–18)** (Figure 2), three new biaryl ethers were obtained from *Phomopsis* sp. of the leaves of *Teucrium scorodonia*. All three compounds were moderately active against *B. megaterium* and *E. coli in vitro*, using 6 mm filter paper disc with 50 μ l each of a 15 mg/mL solution (Krohn et al., 1995). The same compounds were obtained from *Phomosis* sp. of *Ligustrum vulgare* and showed activity against *B. megaterium in vitro* with 10, 10, and 7 mm zone of inhibition using 6 mm filter paper disc and 50 μ g of compound (50 μ L of 1 mg/mL) respectively (Krohn et al., 2011).

Phomosine A (**16**) and Phomosine G (**19**) (Figure 2) were isolated from *Phomopsis* sp. of the halo tolerant plant *Adenocarpus foliolosus* from Gomera. Both the compound exhibited moderate antibacterial activities against *B. megaterium* (Dai et al., 2005).

Phomosine K **(20)**, 2-hydroxymethyl-4β, 5α, 6β-trihydroxycyclohex-2-en **(21)**, (−)-Phyllostine **(22)**, (+)-Epiepoxydon **(23)**, and (+)-Epoxydon monoacetate **(24)** (Figure 2) were isolated from a *Phomopsis* sp. of *Notobasis syriaca.* Phomosine K **(20)** is active against *Legionella pneumophila* Corby, *E. coli* K12 and *B. megaterium in vitro* while 2-hydroxymethyl-4β, 5α, 6β-trihydroxycyclohex-2-en **(21)**, (−)-Phyllostine **(22)**, (+)-Epiepoxydon **(23)**, and (+)-Epoxydon monoacetate **(24)** showed moderate activities against *E. coli* K12 and *B. megaterium* (Hussain et al., 2011).

Phomopsinone B **(25)** and C **(26)** from a *Phomopsis* sp. present in stems of *Santolina chamaecyparissus* from Sardinia showed moderate activities against *E.coli*, and *B. megaterium* (Hussain et al., 2012b).

Phomochromone A (**27**), B (**28**), Phomotenone (**29**), and (1*S*, 2S, 4S)-trihydroxy-*p*-menthane (**30**) (Figure 2) were isolated from a *Phomopsis* sp. of *Cistus monspeliensis*. All three compounds (**27–30**) show activity against *E. coli* and *B. megaterium* (Ahmed et al., 2011).

Pyrenocines J-M (**31–34**) (Figure 2) were isolated from a *Phomopsis* sp. of the plant *Cistus salvifolius*, internal strain 7852. All four compounds (**31–34**) are active against *B. megaterium* and *E. coli* (Hussain et al., 2012a).

3-Nitropropionic acid **(35)** (Figure 2) was isolated from several strains of endophytic fungus of the genus *Phomopsis* sp. obtained from six species of Thai medicinal plants (Table 1) from the forest areas of Chiangmai, Nakhonrachasima, and Pitsanulok Provinces of Thailand. 3-Nitropropionic acid exhibits potent activity against *Mycobacterium tuberculosis* H37Ra with the MIC of 3.3 μ M, but no *in vitro* cytotoxicity was observed toward a number of cell lines (Chomcheon et al., 2005). 3-Nitropropionic acid is known to inhibit isocitrate lyase (ICL), an enzyme required for fatty acid catabolism and virulence in *M. tuberculosis* (Muñoz-Elías and McKinney, 2005).

**Figure 3:**
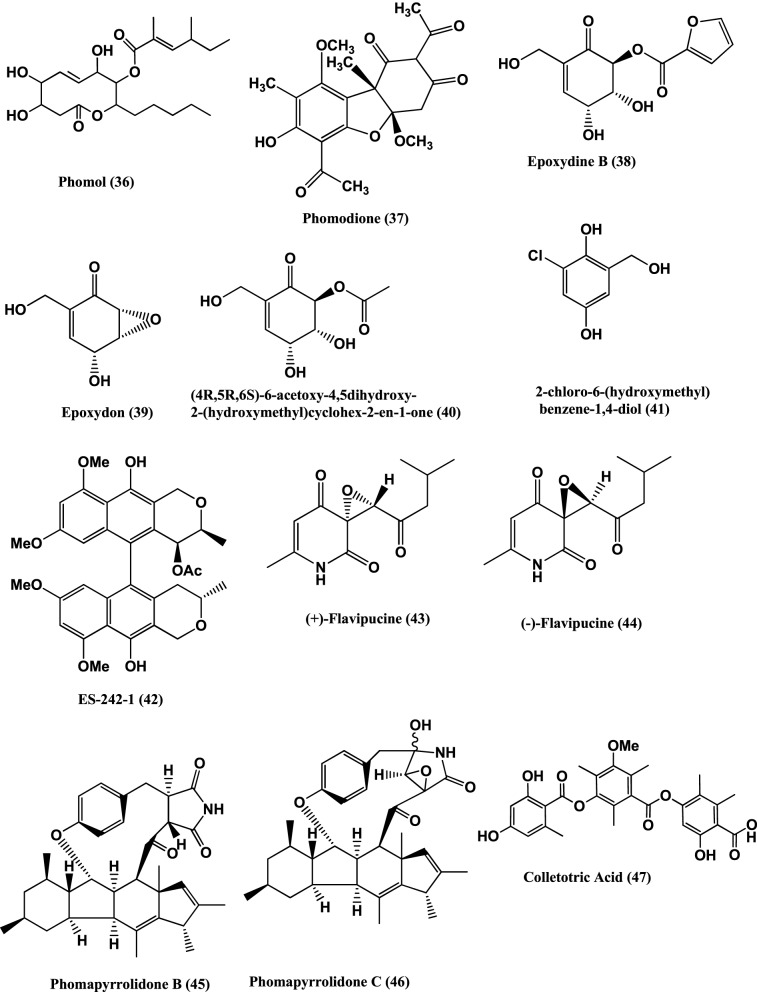
**Structures of antibacterial metabolites isolated from Ascomycetes (36–47)**.

*Phoma* is another genus which produces diverse compounds. Here are some examples of bioactive compounds produced by this genus. Phomol (**36**) (Figure 3), a novel antibiotic, was isolated from a *Phomopsis* sp. of the medicinal plant *Erythrina crista-galli*. Phomol is active against *Arthrobacter citreus* and *Corynebacterium insidiosum* with MICs of 20 and 10 μ g/mL respectively (Weber et al., 2004).

Phomodione **(37)**, an usnic acid derivative was isolated from a *Phoma* sp. of *Saurauia scaberrinae*. Phomodione was found to be effective against *S. aureus* at a MIC of 1.6 μg/mL (Hoffman et al., 2008).

The antibacterials Epoxydine B **(38)**, Epoxydon **(39)**, (4R,5R,6S)-6-acetoxy-4,5-dihydroxy-2-(hydroxymethyl)cyclohex-2-en-1-one **(40)**, 2-chloro-6-(hydroxymethyl)benzene-1,4-diol **(41)**, and the antibiotic ES-242-1 **(42)** (Figure 3), were isolated from a *Phoma* sp. of *Salsola oppostifolia*. Compounds (**38–42**) show activity against *E. coli* and *B. megaterium* (Qin et al., 2010).

Antibacterials (+)-Flavipucine **(43)** and (−)-Flavipucine **(44)** (Figure 3), were isolated from a *Phoma* sp., of the plant *Salsola oppositifolia*. (+)-Flavipucine **(43)** is active against *B. subtilis*, *S. aureus*, *E. coli* with inhibition zones of 16, 17, and 11 mm, respectively in disc diffusion assay at 15 μ g/6 mm. (−)-Flavipucine **(44)** was active against *B. subtilis* and *E. coli* at MIC of 25 μ g/ mL (Loesgen et al., 2011).

Three new alkaloids, Phomapyrrolidones B-C **(45–46)** (Figure 3), were isolated from a *Phoma* sp. NRRL 46751, of the plant *Saurauia scaberrinae*. Phomapyrrolidones B **(45)** and C **(46)** show weak *in vitro* activities when tested in microplate Alamar Blue assays (MABA) with MICs of 5.9 and 5.2 μg/mL respectively and in the low oxygen recovery assay (LORA) with MICs of 15.4 and 13.4 μg/mL respectively, for nonreplicating *M. tuberculosis* H37Pv (Wijeratne et al., 2013).

**Figure 4:**
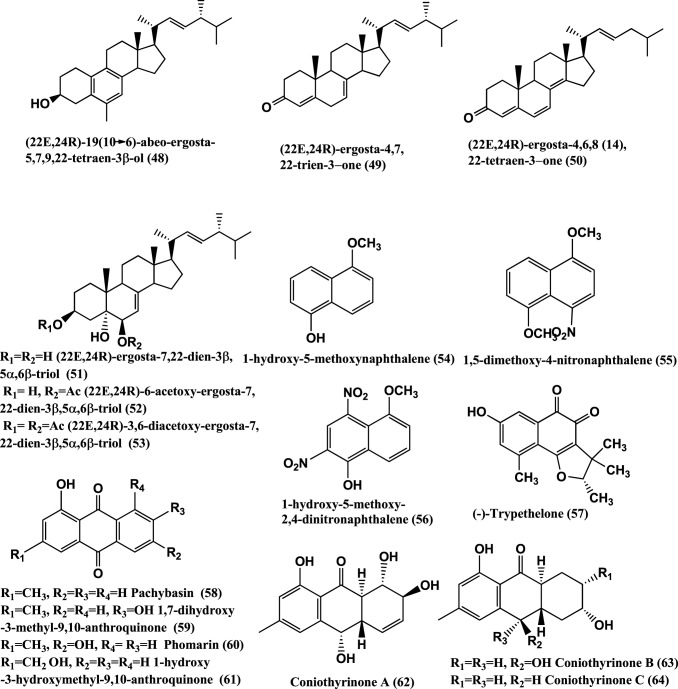
**Structures of antibacterial metabolites isolated from Ascomycetes (48–64)**.

Other endophytes of Ascomycetes are also known to produce antibacterials. For example Colletotric acid **(47)** (Figure 4) from *Colletotrichum gloeosporioides* of *Artemisia mongolica* or Nanjing, China inhibits *B. subtilis*, *S. aureus*, and *Sarcina lutea* with MICs of 25, 50, and 50 μg/mL, respectively (Zou et al., 2000).

Antibacterials (22E,24R)-19(10–>6)-abeo-ergosta-5,7,9,22-tetraen-3β-ol**(48)**, (22E,24R)-ergosta-4,7,22-trien-3-one**(49)**, (22E,24R)-ergosta-4,6,8(14),22-tetraen-3-one **(50)**, (22E,24R)-ergosta-7,22-dien-3β, 5α,6β-triol **(51)**,(22E,24R)-6-acetoxy-ergosta-7,22-dien-3β,5α,6β-triol **(52)**, and (22E,24R)-3,6-diacetoxy-ergosta-7,22-dien-3β,5α,6β-triol **(53)** (Figure 4), were isolated from a *Colletotrichum* sp. of *Ilex canariensis* from Gomera. Compounds **(48–53)** are active against *E. coli* and *B. megaterium* of 0.05 μ g/ filter paper disc of 6 mm diameter (Zhang et al., 2009). Antibacterial 1-hydroxy-5-methoxynaphthalene **(54)**, 1,5-dimethoxy-4-nitronaphthalene **(55)**, 1-hydroxy-5-methoxy-2,4-dinitronaphthalene **(56)** (Figure 4), were isolated from *Coniothyrium* sp. internal strain number 7721 of the shrub *Sideritis chamaedryfolia*, from an arid zone near Alicante, Spain. These compounds were active against *B. megaterium* and *E. coli* (Krohn et al., 2008a).

(−)-Trypethelone **(57)**, isolated from endophyte *Coniothyrium cereale* of the marine green alga *Enteromorpha* sp. showed activity against *Mycobacterium phlei*, *S. aureus*, and *E. coli*, at 20 μ g/disk with inhibition zones of 18, 14, and 12 mm, respectively (Elsebai et al., 2011).

**Figure 5:**
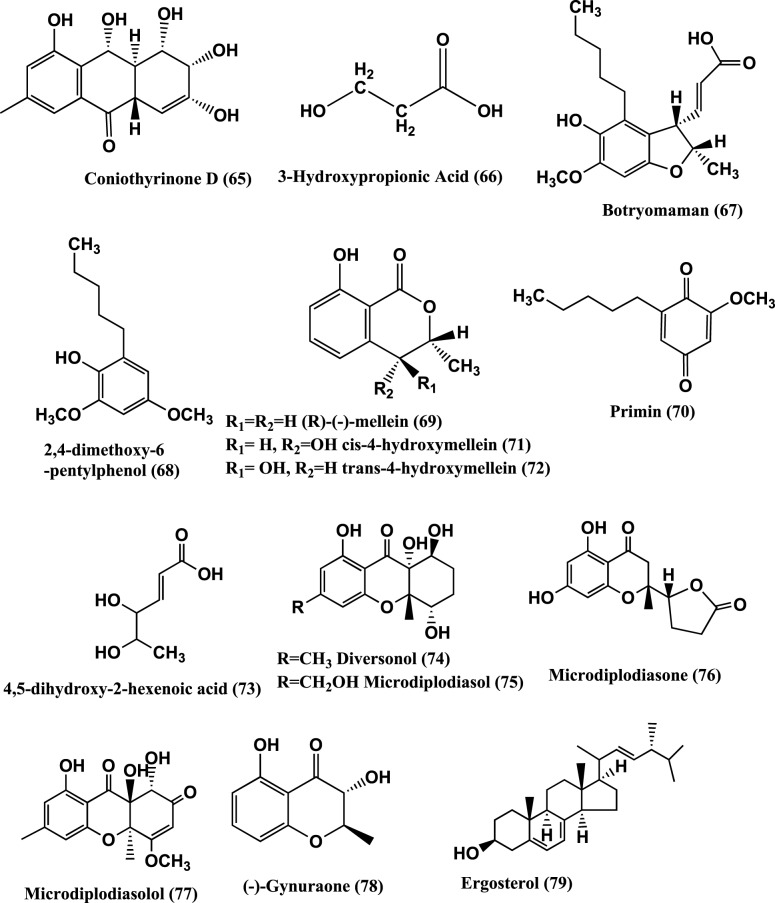
**Structures of antibacterial metabolites isolated from Ascomycetes (65–79)**.

Antibacterials Pachybasin **(58)**, 1, 7-Dihydroxy-3-methyl-9, 10-anthraquinone **(59)**, Phomarin **(60)**, 1-Hydroxy-3-hydroxymethyl-9, 10-Anthraquinone (**61**), and Coniothyrinones A-D **(62–65)** (Figures 4, 5), were isolated from *Coniothyrium* sp., an endophyte of *Salsola oppostifolia* from Gomera in the Canary Islands. Compounds **(58–65)** were active against *E. coli* and *B. megaterium in vitro* in disc diffusion assay at 50 μ g/9 mm disc dissolved in acetone (Sun et al., 2013a).

3-Hydroxypropionic acid (3-HPA) **(66)** (Figure 5) was isolated from the mangrove endophyte *Diaporthe phaseolorum*, from branches of *Laguncularia racemosa*, growing in Bertioga, located in south eastern Brazil. 3-HPA was active against both *S. aureus* and *Salmonella typhi* at an MIC of 64 μ g/mL (Sebastianes et al., 2012).

Botryomaman **(67)**, 2, 4-Dimethoxy-6-pentylphenol **(68)**, (*R*)—(−)-Mellein **(69)**, Primin **(70)**, *cis*-4-hydroxymellein **(71)**, *trans*-4-hydroxymellein **(72)** and 4, 5-dihydroxy-2-hexenoic acid **(73)** (Figure 5) were isolated from the endophyte *Botryosphaeria mamane* PSU-M76 from the leaves of *Garcinia mangostana*, collected in Suratthani Province, Thailand. The compounds were active against *S. aureus* ATCC 25923 and MRSA SK1. Primin was the most active with MIC values of 8 μ g/mL against both the strains (Pongcharoen et al., 2007).

*Microdiplodia* sp. isolated from the shrub *Lycium intricatum* gave Diversonol (**74**), Microdiplodiasol (**75**), Microdiplodiasone (**76**), Microdiplodiasolol (**77**), (−)-Gynuraone (**78**), and Ergosterol (**79**) (Figure 5). Compounds **(74–79)** were active against *Legionella pneumophila* (Siddiqui et al., 2011).

**Figure 6:**
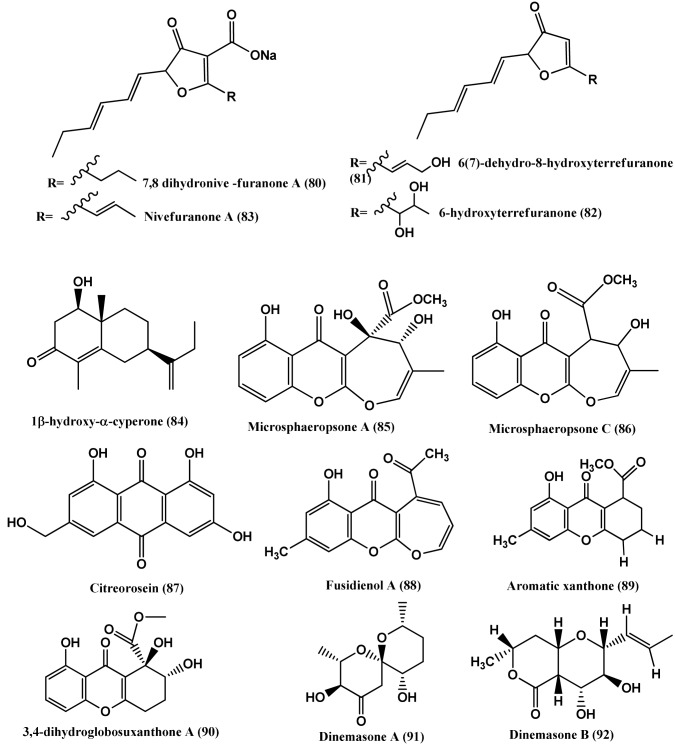
**Structures of antibacterial metabolites isolated from Ascomycetes (80–92)**.

Polyketide metabolites, 7,8-dihydonivefuranone A **(80)**, 6(7)-dehydro-8-hydroxyterrefuranone **(81)**, 6-hydroxyterrefuranone **(82)** and Nivefuranones A **(83)** (Figure 6) were isolated from a *Microdiplodia* sp. KS 75-1 from the stems of conifer trees (*Pinus* sp.). Compounds **(80–83)** were active against *S. aureus* NBRC 13276 with zone of inhibition of 15, 15, 16, and 15 mm respectively, tested at 40 μ g/per disc of 8 mm diameter (Shiono et al., 2012).

1β-hydroxy-α-cyperone **(84)** (Figure 6) was isolated from the endophyte *Microsphaeropsis arundinis* found in stems of *Ulmus macrocarpa* collected from Dongling Mountain, Beijing, People's Republic of China. Compound **(84)** inhibits *S. aureus* (CGMCC1.2465), at an MIC of 11.4 μ g/mL. Ampicillin (positive control) showed an MIC value of 0.46 μ g/mL (Luo et al., 2013).

Microsphaeropsone A **(85)** and Microsphaeropsone C **(86)** (Figure 5), were isolated from *Microsphaeropsis* sp. (strain 8875) from the plant *Lycium intricatum*, co-occurs with their putative biogenetic Anthraquinoide precursors and Citreorosein **(87)**. From a *Microsphaeropsis* species (strain no. 7177) of the plant *Zygophyllum fortanesii* from Gomera (Spain), large amounts of Fusidienol A **(88)** and the known aromatic xanthones **(89)**, were isolated. The endophyte *Seimatosporium* species (internal strain no. 8883) of *Salsola oppositifolia* from Gomera (Spain), produced 3, 4-dihydroglobosuxanthone A **(90)**. Compounds **(85–90)** were active against *E. coli* and *B. megaterium* (Krohn et al., 2009).

Dinemasones A**(91)** and B **(92)** (Figure 5), were isolated from *Dinemasporium strigosum* obtained from the roots of the herbaceous plant *Calystegia sepium* growing on the shores of the Baltic Sea, Wustrow, Germany. The above compounds showed antibacterial activities against *B. megaterium* (Krohn et al., 2008b).

**Figure 7:**
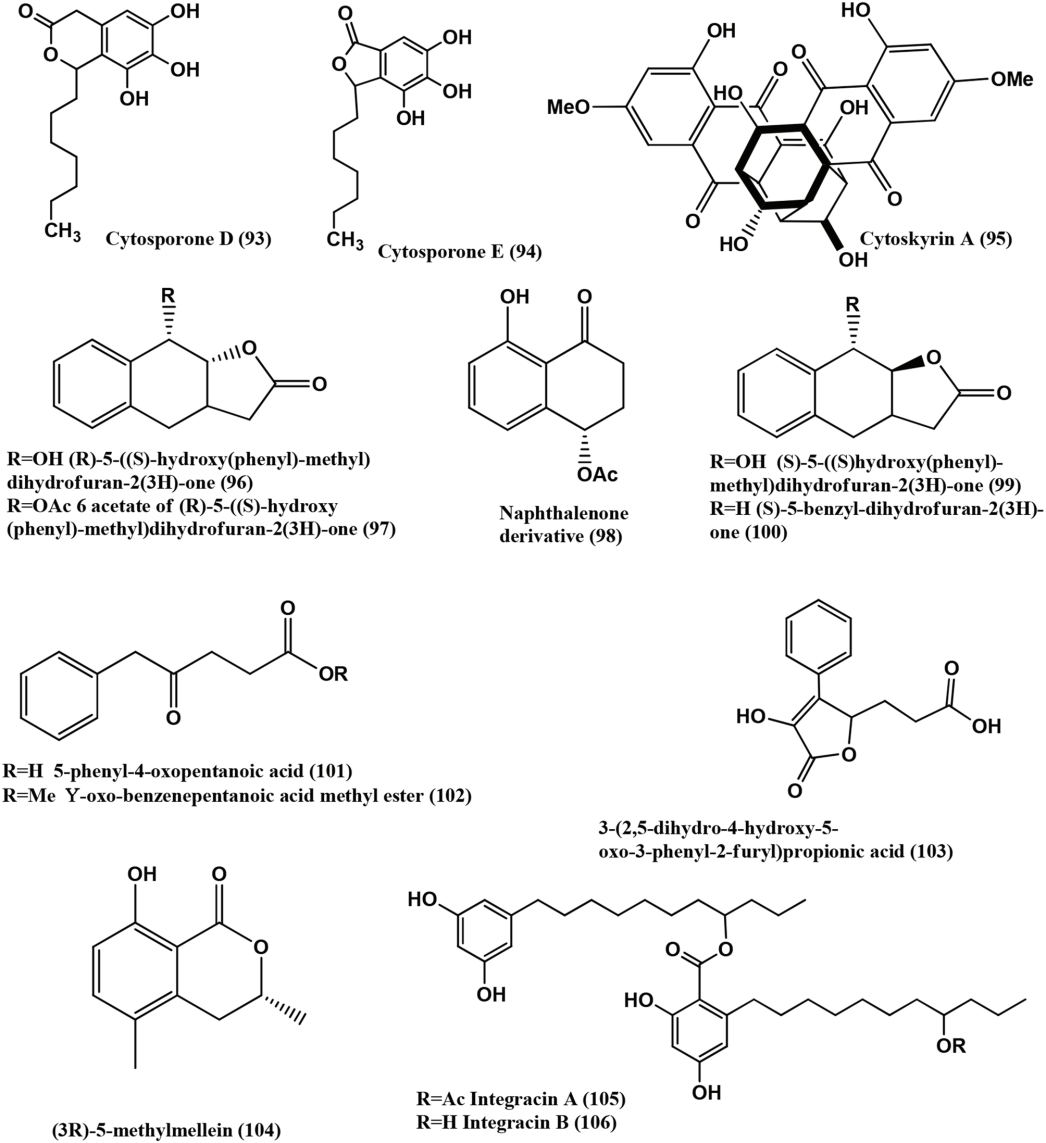
**Structures of antibacterial metabolites isolated from Ascomycetes (93–106)**.

Cytosporone D **(93)** and E **(94)** (Figure 7), were isolated from the endophyte CR200 (*Cytospora* sp.) and CR146 (*Diaporthe* sp.) present in tissues of *Conocarpus erecta* and *Forsteronia spicata* plants respectively collected in the Guanacaste Conservation Area of Costa Rica. Cytosporone D **(93)** shows antibacterial activity against *S. aureus, E. faecalis*, and *E. coli* with MICs of 8, 8, and 64 μ g/mL respectively, while Cytosporones E **(94)** has similar activity against *S. aureus* (Brady et al., 2000).

Cytosporone D **(93)**, E **(94)**, and Cytoskyrin A **(95)** (Figure 7), were isolated from a *Cytospora* sp. CR200 from a branch of *Conocarpus erecta* (Buttonwood tree) in the Guanacaste National Park, from Costa Rica. Cytoskyrin A **(95)** has good *in-vitro* antibacterial activity (MICs against (*S. aureus* ATCC 29923, *S. aureus* ATCC6538P, *S. aureus* #310 (MRSA), *E. faecium* #379 (VREF), *E. faecium* # 436 (VSEF), *B. subtilis* BGGS1A1, *E. coli imp* BAS849), ranging from 0.03 to 0.25 μ g/mL). Cytosporone D **(93)** and E **(94)** have moderate *in-vitro* antibacterial activity against above mentioned bacteria (MICs 8–64 μ g/mL) (Singh et al., 2007).

Two new benzyl γ-butyrolactone analogs, (R)-5-((S)-hydroxy(phenyl)-methyl)dihydrofuran-2(3H)-one **(96)** and its 6-acetate **(97)**, a new naphthalenone derivative **(98)**, together with aromatic derivatives, (S)-5-((S)-hydroxy(phenyl)-methyl)dihydrofuran-2(3H)-one **(99)**, (S)-5-benzyl-dihydrofuran-2(3H)-one **(100)**, 5-phenyl-4-oxopentanoic acid **(101)**, gamma-oxo-benzenepentanoic acid methyl ester **(102)**, 3-(2,5-dihydro-4-hydroxy-5-oxo-3-phenyl-2-furyl)propionic acid **(103)**, (3R)-5-methylmellein **(104)**, Integracins A **(105)**, and B **(106)** (Figure 7) were isolated from *Cytospora* sp., of *Ilex canariensis* from Gomera. Compounds **(96- 106)** are active against *B. megaterium*, zone size range 15–25 mm when 50 μ L of a solution (0.05 mg/mL substance) are pipetted onto 9 mm a sterile filter paper disc (Lu et al., 2011).

**Figure 8:**
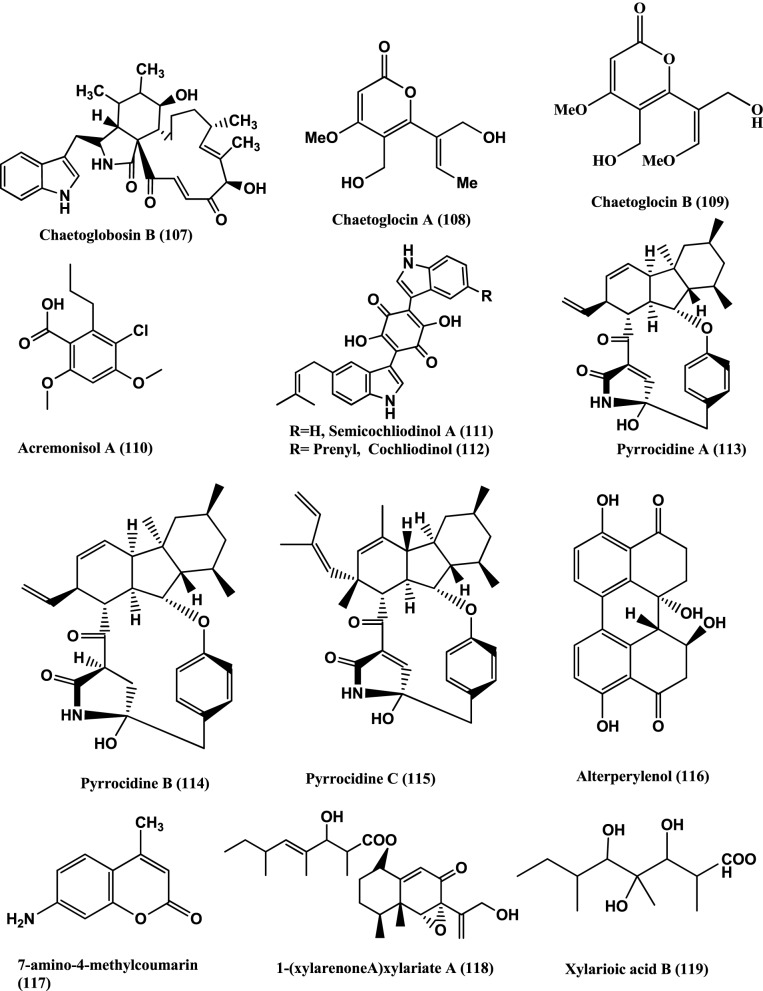
**Structures of antibacterial metabolites isolated from Ascomycetes (107–119)**.

Chaetoglobosin B **(107)** (Figure 8), isolated from the endophyte *Chaetomium globosum* from the leaves of *Viguiera robusta* showed weak antibacterial activity against *S. aureus* (MIC 120 μ g/mL) and *E. coli* (MIC 189 μ g/mL) (Momesso et al., 2008).

Chaetoglocins A-B **(108–109)** (Figure 8) isolated from *Chaetomium globosum* strain IFB-E036, an endophyte from *Cynodon doctylon* have antimicrobial activity against *B. subtilis*, *Streptococcus pyogens*, *Micrococcus luteus* and *Mycobacterium smegmatis* with MICs between 8 and 32 μ g/mL (Ge et al., 2011).

Antibacterial compounds Acremonisol A **(110)**, Semicochliodinol A **(111)**, Cochliodinol **(112)**, were isolated from *C. globosum* SNB-GTC2114 and Pyrrocidine A **(113)**, B **(114)**, C **(115)**, and Alterperylenol **(116)** (Figure 8) were isolated from *Lewia infectoria* SNB-GTC2402 obtained from *Besleria insolita* from the Amazon Rainforest biome of Cayenne and Roura, French Guiana. Compounds **(110–112**, **115**, and **116**), exhibited antibacterial activity against *S. aureus* ATCC 29213 with MICs of 64, 2, 4, 2, and 32 μg/mL respectively. Compounds (**113–114**) were active against *S. aureus* ATCC 29213, with a MIC value of 5 μ g/mL (Casella et al., 2013).

7-amino-4-methylcoumarin **(117)** (Figure 8) was isolated from the endophyte *Xylaria* sp., of *Ginkgo biloba*. The compound showed strong antibacterial against *S. aureus, E. coli, S. typhi, Salmonella typhimurium, Salmonella enteritidis, Aeromonas hydrophila, Yersinia* sp., *Vibrio anguillarum, Shigella* sp., and *Vibrio parahaemolyticus* with MIC of 16, 10, 20, 15, 8.5, 4, 12.5, 25, 6.3, and 12.5 μ g/mL respectively (Liu et al., 2008).

**Figure 9:**
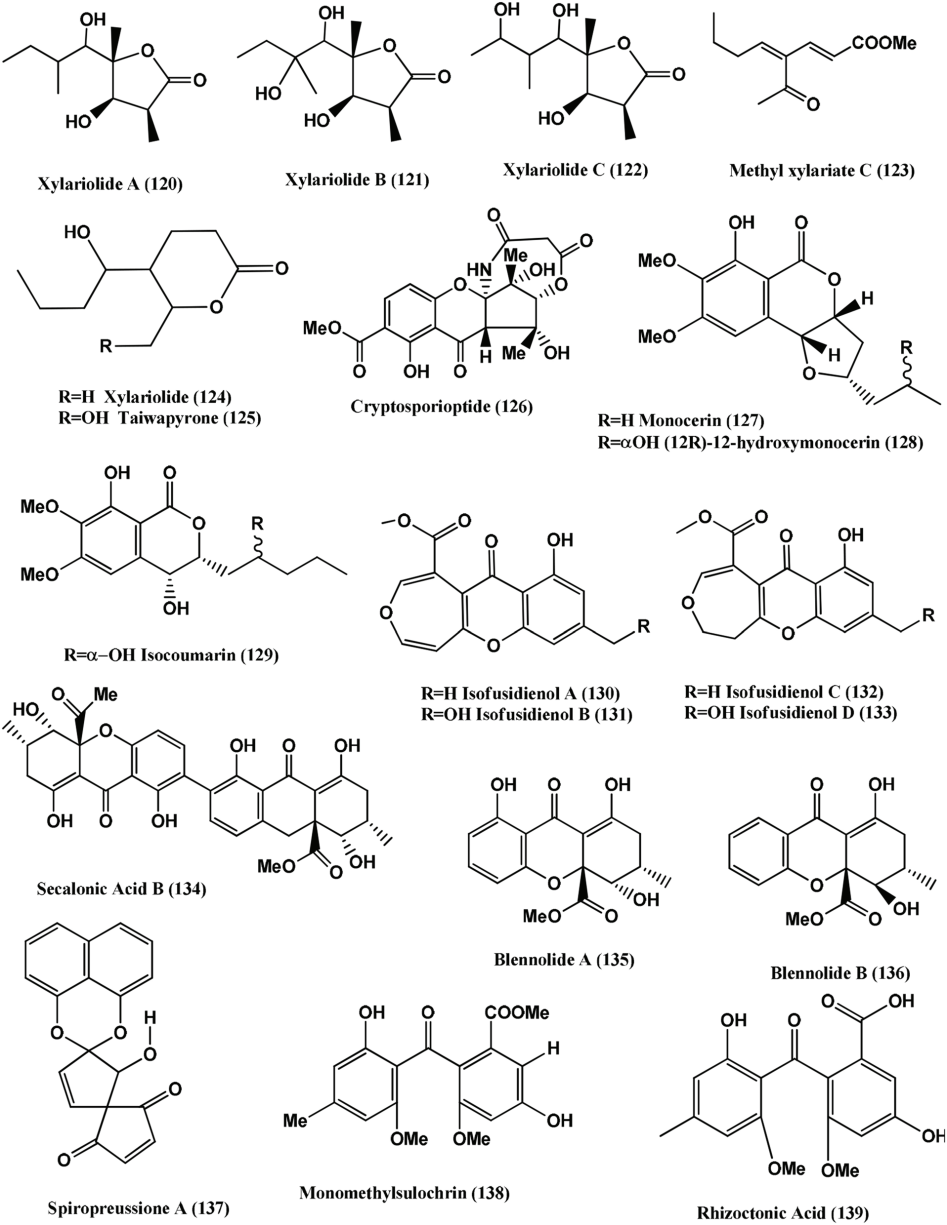
**Structures of antibacterial metabolites isolated from Ascomycetes (120–139)**.

1-(xylarenone A)xylariate A **(118)**, Xylarioic acid B **(119)** (Figure 8), Xylariolide A **(120)**, Xylariolide B **(121)**, Xylariolide C **(122)**, Me-xylariate C **(123)**, Xylariolide D **(124)**, and taiwapyrone **(125)** (Figure 9), were isolated from *Xylaria* sp. NCY2 of *Torreya jackii* Chun collected from Jiangshi Nature Reserve Zone of Fujian Province, China. Compounds **(118–125)** are active against *E. coli* ATCC 25922, *B. subtilis* ATCC 9372 and *S. aureus* ATCC 25923 with MIC values above 10 μ g/mL (Hu et al., 2010).

The polyketide, Cryptosporioptide **(126)** (Figure 9) was isolated from a *Cryptosporiopsis* sp., from the shoot tissues of the shrub *Viburnum tinus*, collected from Gomera. At 50 μ g per 9 mm paper disc, it inhibits *B. megaterium*, showing a 9 mm radius of zone of inhibition (Saleem et al., 2013).

Monocerin **(127)**, (12*S*)-12-hydroxymonocerin **(128)** and Isocoumarin **(129)** (Figure 9) were isolated from *Microdochium bolleyi*, an endophyte from *Fagonia cretica.* All these compounds were active against *E. coli* and *B. megaterium* (Zhang et al., 2008a).

Isofusidienol A **(130)**, B **(131)**, C **(132)**, and D **(133)** (Figure 9) were isolated from a *Chalara* sp. strain 6661, an endophyte of *Artemisia vulgaris*, collected from Ahrenshoop, Germany Compounds **(130)** and **(131)** showed strong antibacterial activities against *B. subtilis* with inhibition zones of 23 and 22 mm respectively, at 15 μ g of compounds per 6-mm filter disks. Under the same conditions, 15 μ g of Penicillin G has a zone of 50-mm diameter. The MIC of compound **(130)** was shown to be 0.625 μ g on 6-mm filter disks. Compound **(130)** shows moderate activity against *S. aureus* and *E. coli* with an inhibition zone diameter of 9 and 8 mm, respectively, at 15 μ g of compound per 6-mm filter disk. Compound **(132)** and **(133)** show inhibition zone of 9 and 8 mm against *B. subtilis* at 15 μ g per 6-mm filter disk (Loesgen et al., 2008).

Secalonic acid B **(134)**, Blennolides A **(135)** and B **(136)** (Figure 9) were isolated from a *Blennoria* sp., an endophyte of *Carpobrotus edulis*, from El Cedro, Gomera. Compounds **(134–136)** inhibit *B. megaterium*, and compounds **(135)** and **(136)** also inhibited *E. coli* (Zhang et al., 2008b).

Spiropreussione A **(137)** (Figure 9) was obtained from an endophyte, *Preussia* sp., of the mature stems of *Aquilaria sinensis* (Thymelaeaceae), collected from the Guangxi Medicinal Arboretum. Spiropreussione A **(137)** shows activity against *S. aureus* (CMCC B26003) with a zone of inhibition of 16.4 ± 0.3 mm (*n* = 3) at 5 μ g/disk. The MIC of the compound in agar dilution test using NCCLS 2002 guide lines was 25 μ M (Chen et al., 2009).

**Figure 10:**
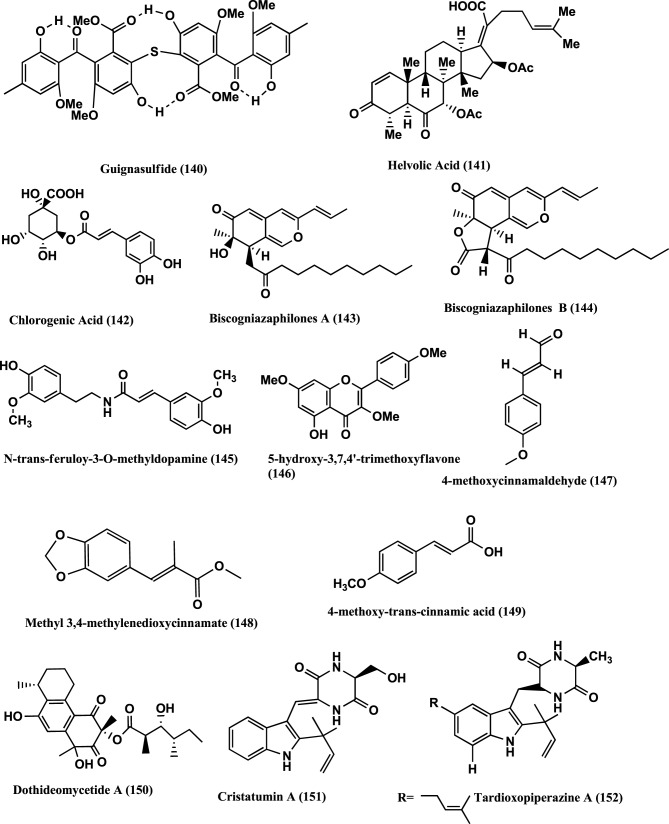
**Structures of antibacterial metabolites isolated from Ascomycetes (140–152)**.

Monomethylsulochrin **(138)**, Rhizoctonic acid **(139)**, (Figure 9) and Guignasulfide **(140)** (Figure 10) were isolated from a *Guignardia* sp. IFB-E028, an endophyte of *Hopea hainanensis* and show moderate activity against the human bacterial pathogen *Helicobacter pylori* with MIC values of 28.9, 60.2, and 42.9 μM, respectively (Wang et al., 2010).

Helvolic acid **(141)** (Figure 10) was isolated from the endophyte *Pichia guilliermondii* Ppf9 of medicinal plant *Paris polyphylla* var. *yunnanensis*. Compound **(141)** has strongest antibacterial activity on *Agrobacterium tumefaciens*, *E. coli, Pseudomonas lachrymans*, *Ralstonia solanacearum*, *Xanthomonas vesicatoria*, *B. subtilis*, *S. aureus*, and *Staphylococcus haemolyticus*, with MICs of 1.56, 3.13, 3.13, 1.56, 1.56, 3.13, 50, and 6.25 μ g/mL, respectively (Zhao et al., 2010).

Chlorogenic acid **(142)** (Figure 10) was isolated from the endophyte strain B5 a *Sordariomycete* sp. of *Eucommia ulmoides*. *Eucommia ulmoides* is a medicinal plant of China and one of the main sources of Chlorogenic acid. It has antibacterial, antifungal, antioxidant and antitumor activities (Chen et al., 2010).

Antibacterial Biscogniazaphilones A **(143)** and B **(144)**, N-trans-feruloy-3-O-methyldopamine **(145)**, 5-Hydroxy-3,7,4-trimethoxyflavone **(146)**, 4-Methoxycinnamaldehyde **(147)**, Methyl 3,4-methylenedioxycinnamate **(148)**, 4-Methoxy-trans-cinnamic acid **(149)**, (Figure 10) were isolated from the endophyte *Biscogniauxia formosana* BCRC 33718, of *Cinnamomum* sp. Compounds **(143)** and **(144)** show antimycobacterial activities against *M. tuberculosis* strain H37Rv *in vitro* showing MIC values of ≤5.12 and ≤2.52 μ g/mL, respectively, than the clinical drug Ethambutol (MIC 6.25 μ g/mL). Compounds **(145–149)** show moderate to weak antimycobacterial activities with MICs of 12.5, 25.0, 42.1, 58.2, and 50.0 μ g/mL, respectively (Cheng et al., 2012).

Dothideomycetide A **(150)** (Figure 10) from an endophyte a *Dothideomycete* sp., of a Thai medicinal plant, *Tiliacora triandra*, has antibacterial activity against *S. aureus* ATCC 25923 and MRSA ATCC 33591 with MIC values of 128 and 256 μ g/ mL respectively (Senadeera et al., 2012).

Cristatumins A **(151)** and Tardioxopiperazine A **(152)** (Figure 10) were produced by the endophyte *Eurotium cristatum* EN-220 of marine alga *Sargassum thunbergii* and showed activity against *E. coli* and *S. aureus* with MIC values of 64 and 8 μ g/mL, respectively (Du et al., 2012).

### Compounds produced by hyphomycetes

**Figure 11:**
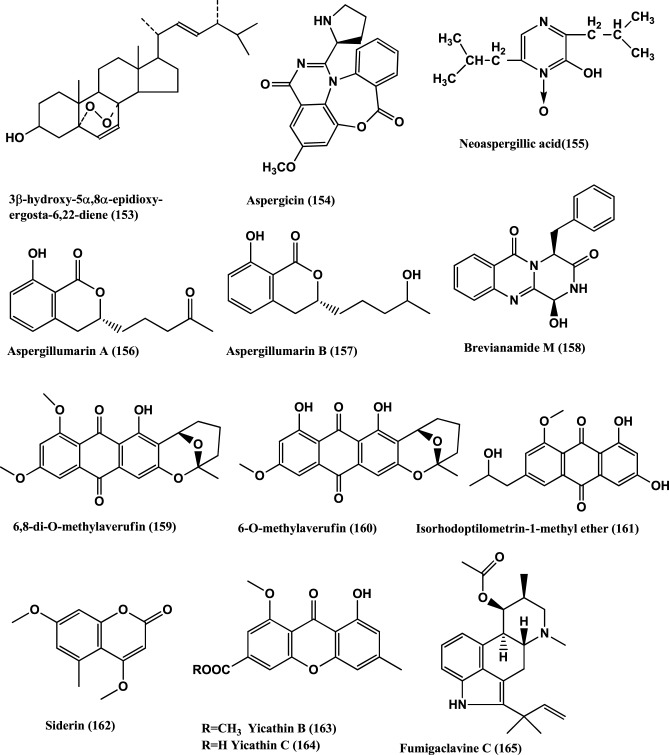
**Structures of antibacterial metabolites isolated from Hyphomycetes (153–165)**.

Hyphomycete form a class of fungi which produces the asexual spores. Producers of the antibacterials Penicillins and Cephalosporins belong to this class. Other antibacterials from this class are Helvolic acid **(141)** (Figure 10), Monomethylsulochrin **(138)** (Figure 9), Ergosterol **(79)** (Figure 5) and 3β-Hydroxy-5α, 8α-epidioxy-ergosta-6, 22-diene **(153)** (Figure 11) were isolated from an endophyte *Aspergillus* sp. CY725 of *Cynodon dactylon* (Poaceae). Compounds **(141)**, **(138)**, **(79)**, and **(153)** are active against *H. pylori* with MICs of 8.0, 10.0, 20.0, and 30.0 μ g/mL respectively. Helvolic acid **(141)** is active against *Sarcina lutea* and *S. aureus* with MICs of 15.0 and 20.0 μ g/mL respectively (Li et al., 2005).

Aspergicin **(154)** and Neoaspergillic acid **(155)** (Figure 11) were isolated from a mixture of cultured mycelia of two marine-derived mangrove epiphytic *Aspergilli* FSY-01 and FSW-02. Aspergicin **(154)** has anti-bacterial activity against *S. aureus*, *S. epidermidis*, *B. subtilis*, *B. dysenteriae, B. proteus*, and *E. coli*, with MICs of 62.5, 31.25 15.62, 15.62 62.5, and 31.25 μ g/mL respectively. Neoaspergillic acid **(155)** has antibacterial activity against *S. aureus*, *S. epidermidis*, *B. subtilis*, *B. dysenteriae, B. proteus*, and *E. coli*, with MICs of 0.98, 0.49, 1.95, 7.8, 7.8, and 15.62 μ g/mL respectively (Zhu et al., 2011).

Two new dihydroisocoumarin derivatives Aspergillumarins A **(156)** and B **(157)** (Figure 11) are produced by a marine-derived *Aspergillus* sp., of the mangrove *Bruguiera gymnorrhiza* collected from the South China Sea. Both show weak antibacterial activities against *S. aureus* and *B. subtilis* at 50 μ g/mL (Li et al., 2012).

Brevianamide M **(158)**, 6, 8-di-O-methylaverufin **(159)** and 6-O-Methylaverufin **(160)** (Figure 11), were isolated from *Aspergillus versicolor* a fungus of the marine brown alga *Sargassum thunbergii.* These compounds have activities against *S. aureus* and *E. coli* (Miao et al., 2012).

Isorhodoptilometrin-1-Me ether **(161)**, Siderin **(162)** (Figure 11), were isolated from the marine fungus *Aspergillus versicolor* of inner tissues of the Red Sea green alga *Halimeda opuntia*. Both the compounds show moderate activity against *Bacillus cereus, B. subtilis*, and *S. aureus* at a concentration of 50 μg/disc of 9 mm (Hawas et al., 2012).

Yicathin B **(163)** and C **(164)** (Figure 11) were isolated from the endophyte *Aspergillus wentii* PT-1 of the red marine alga *Gymnogongrus flabelliformis*. Tested in the agar diffusion assay at 10 mg/disk compound **(163)** was active against *E. coli* (inhibition zone diameter 9 mm) and **(164)** a zone diameter of 12.0 mm and against *S. aureus* 7.5 mm (Sun et al., 2013b).

**Figure 12:**
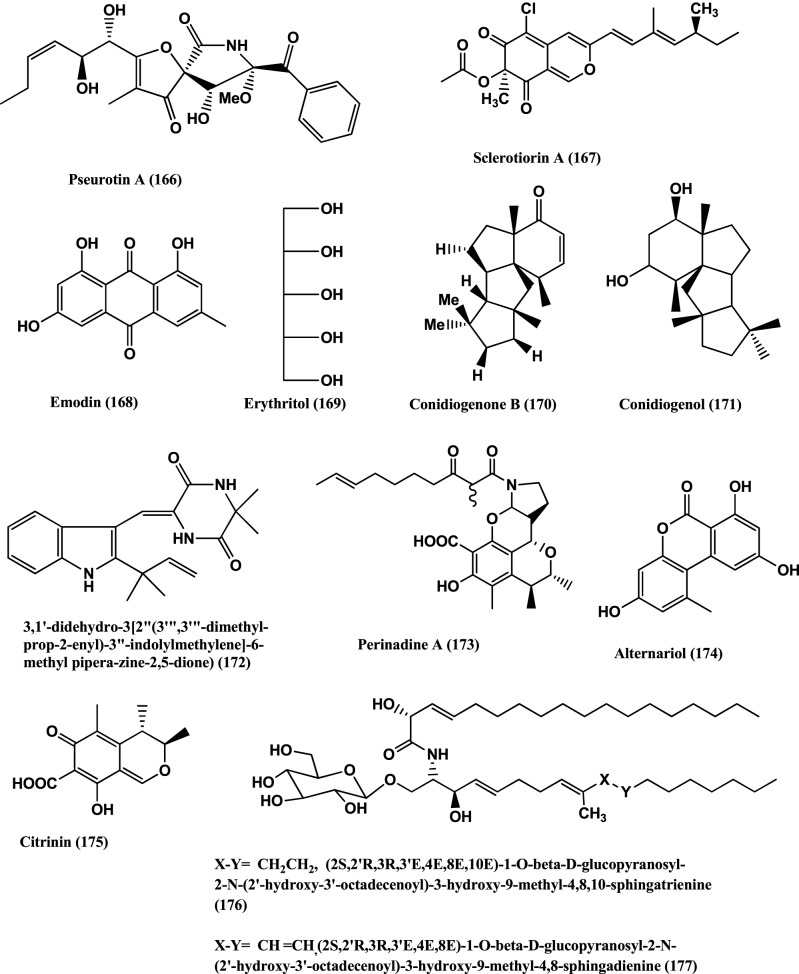
**Structures of antibacterial metabolites isolated from Hyphomycetes (166–177)**.

The alkaloids, Fumigaclavine C **(165)** (Figure 11) and Pseurotin A **(166)** (Figure 12) were isolated from the endophyte *Aspergillus* sp. EJC08, of the medical plant *Bauhinia guianensis.* Fumigaclavine C **(165)** has activity against *B. subtilis*, *E. coli*, *P. aeruginosa*, and *S. aureus* with MICs of 7.81, 62.50, 31.25, and 15.62 μ g/mL respectively, while Pseurotin A **(166)** has activity against *B. subtilis, E. coli, P. aeruginosa*, and *S. aureus* with MICs of 15.62, 31.25, 31.25, and 15.62 μ g/mL respectively (Pinheiro et al., 2013).

Pseurotin A **(166)** (Figure 12) was isolated from *Penicillium janczewskii* of the Chilean gymnosperm *Prumnopitys andina*. The compound shows moderate activity against phytopathogenic bacteria *Erwinia carotovora* and *Pseudomonas syringae*, with IC_50_ values of 220 and 112 μ g/ mL, respectively (Schmeda-Hirschmann et al., 2008).

(+)-Sclerotiorin **(167)** (Figure 12), was isolated from the endophyte *Penicillium sclerotiorum* PSU-A13 (Arunpanichlert et al., 2010). Compound **(167)** has been reported to have antibacterial activity against *S. aureus* ATCC 29213 (MIC 128 μg/mL) (Lucas et al., 2007).

Emodin **(168)** and Erythritol **(169)** (Figure 12) were isolated from the endophyte *Penicillium citrinum* strain ZD6 of the stems of *Bruguiera gymnorrhiza.* Emodin **(168)** and Erythritol **(169)** inhibit the growth of *B. subtilis* with MIC values of 25 μ g/mL and 50 μ g/mL respectively, while Emodin **(168)** was weakly active against *P. aeruginosa* at an MIC value of 100 μ g/mL (Li et al., 2010).

Antibacterial Conidiogenone B **(170)** and Conidiogenol **(171)** (Figure 12) were isolated from *Penicillium chrysogenum* QEN-24S, an endophyte of a marine red algal species of the genus *Laurencia*. Conidiogenone B **(170)** has potent activity against MRSA, *Pseudomonas fluorescens*, P. *aeruginosa*, and *S. epidermidis* (at a concentration of 8 μ g/mL), while Conidiogenol **(171)** is activity against *P. fluorescens* and *S. epidermidis* (both at an MIC value of 16 μ g/mL) (Gao et al., 2011).

(3, 1′-didehydro-3[2″ (3‴, 3‴;-dimethyl-prop-2-enyl)-3″-indolylmethylene]-6-Mepipera-zine-2, 5-dione) **(172)** (Figure 12) was isolated from *Penicillium chrysogenum* MTCC 5108, an endophyte of the mangrove plant *Porteresia coarctata* (Roxb.), which has significant activity against *Vibrio cholera* MCM B-322 (Devi et al., 2012).

Perinadine A **(173)**, Alternariol **(174)**, and Citrinin **(175)** (Figure 12) were isolated from *Penicillium citrinum* present on the flowers of *Ocimum tenuiflorum* (Lamiaceae) collected in Denpasar, Bali, Indonesia. Compounds (**173–175)** were moderately active against *S. aureus* ATCC 29213 (MICs 64 μ g/mL). These compounds, failed to inhibit the *E. coli* ATCC 25922, and *P. aeruginosa* B 63230 at 64 μ g/mL (Lai et al., 2013).

Fusarusides (2S,2′R,3R,3′E,4E,8E,10E)-1-O-β-D-glucopy-ranosyl-2-N-(2′-hydroxy-3′-octadecenoyl)-3-hydroxy-9-methyl-4,8,10-sphingatrienine **(176)**, (2S,2′R,3R,3′E,4E,8E)-1-O-β-D-glucopyranosyl-2-N-(2′-hydroxy-3′-octadecenoyl)-3-hydroxy-9-methyl-4,8-sphingadienine **(177)** (Figure 12) were isolated from a *Fusarium* sp. IFB-121, an endophyte of *Quercus variabilis.* Both cerebrosides have strong antibacterial activities against *B. subtilis*, *E. coli* and *P. fluorescens* with MIC values of 3.9, 3.9 and 1.9 μ g/mL and 7.8, 3.9, and 7.8 μ g/mL respectively (Shu et al., 2004).

**Figure 13:**
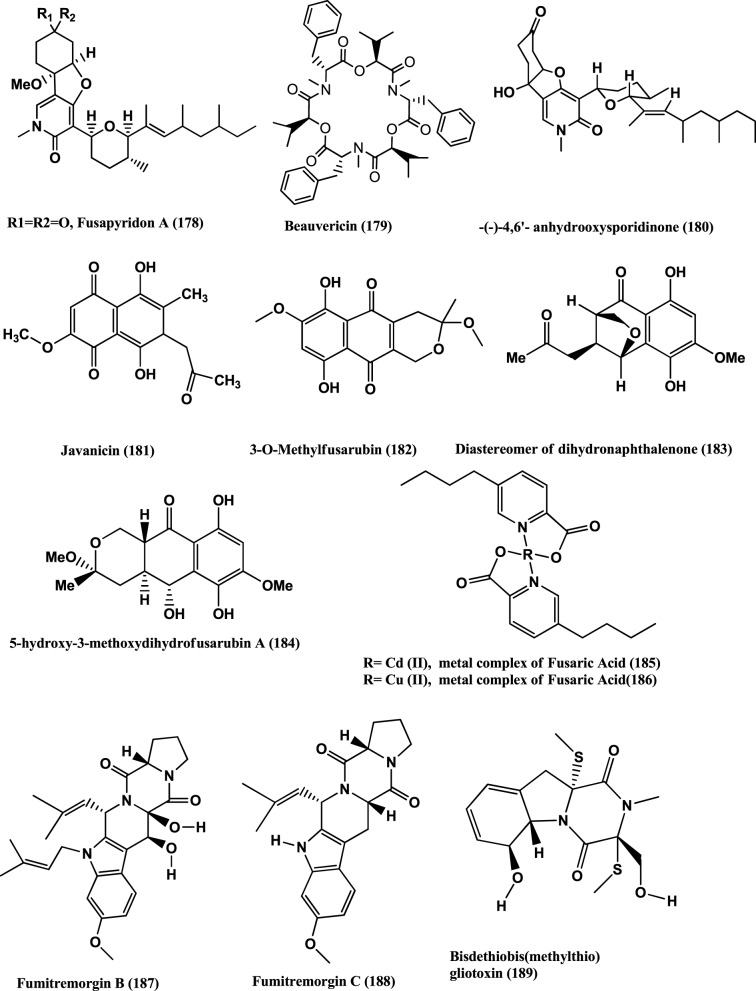
**Structures of antibacterial metabolites isolated from Hyphomycetes (178–189)**.

Fusapyridon A **(178)** (Figure 13) was isolated from *Fusarium* sp. YG-45, an endophyte of the stem of *Maackia chinensis*, collected at Gottingen (Germany). The compound is active against *P. aeruginosa* and *S. aureus*, with MIC values of 6.25 and 50 μ g/mL, respectively (Tsuchinari et al., 2007).

Beauvericin **(179)** (Figure 13) was found in the endophyte *Fusarium redolens* Dzf2, of the rhizomes of *Dioscorea zingiberensis*. The IC_50_ values of Beauvericin against six test bacteria viz. *B. subtilis, Staphylococcus hemolyticus, Pseudomonas lachrymans, Agrobacterium tumefaciens, E. coli* and *X. vesicatoria* were between 18.4 and 70.7 μ g/mL (Xu et al., 2010b). Beauvercin and (−)-4, 6′-anhydro-oxysporidinone **(180)** (Figure 13) were isolated from the endophyte *Fusarium oxysporum* of the bark of *Cinnamomum kanehirae* from Jiaoban Mountain, Taiwan Province. Beauvericin **(179)** is active against MRSA and *B. subtilis* at MICs of 3.125 μ g/mL. (−)-4, 6′-anhydro-oxysporidinone **(180)** has weak anti-MRSA activity (MIC, 100 μ g/mL) and moderate activity against *B. subtilis* (MIC, 25 μ g/mL) (Wang et al., 2011).

Javanicin **(181)**, 3-O-methylfusarubin **(182)**, a diastereomer of Dihydronaphthalenone **(183)** and 5-Hydroxy-3-methoxydihydrofusarubin A **(184)** (Figure 13) were isolated from the endophyte *Fusarium* sp. BCC14842 of Bamboo leaf, collected from the Bamboo forest at Nam Nao National Park, Phetchabun Province, Thailand. Compound **(181)**, and **(183)** have moderate activities (MICs of 25 μ g/mL) while 3-O-methylfusarubin **(182)** and 5-hydroxy-3-methoxydihydrofusarubin A **(184)** showed weak antimycobacterial activity (MICs of 50 μ g/mL) (Kornsakulkarn et al., 2011).

Fusaric acid was obtained from a *Fusarium* sp. an endophyte of a mangrove plant. Cadmium and Copper metal complexes were prepared. The Cadmium **(185)** and Copper **(186)** (Figure 13) complexes of fusaric acid exhibited potent inhibitory activity against the *Mycobacterium bovis* BCG strain with MIC 4 μ g/mL and the *M. tuberculosis* H37Rv strain with MIC 10 μ g/mL respectively (Pan et al., 2011).

**Figure 14:**
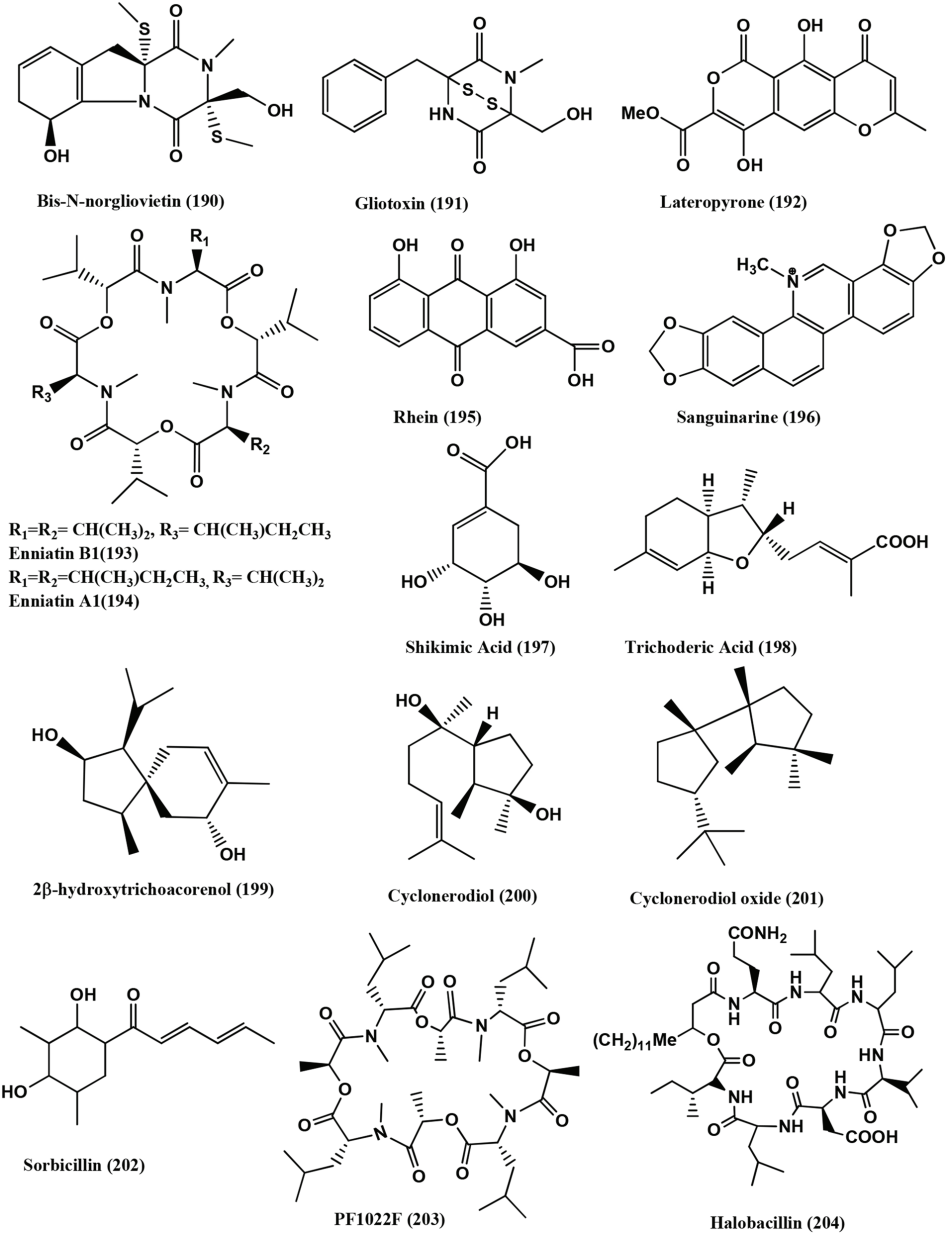
**Structures of antibacterial metabolites isolated from Hyphomycetes (190–204)**.

Fumitremorgin B **(187)**, Fumitremorgin C **(188)**, Helvolic acid **(141)**, Bisdethiobis (methylthio) gliotoxin **(189)** (Figure 13), Bis-N-norgliovietin **(190)** and Gliotoxin **(191)** (Figure 14) were isolated from the endophyte *Fusarium solani* of *Ficus carica*. All compounds are active against B*. subtilis*, *S. aureus*, and *E. coli* and *P. aeruginosa* with MICs in the range of 0.5–16 μg/mL (Zhang et al., 2012).

Lateropyrone **(192)**, Enniatins B1 **(193)** and A1 **(194)** (Figure 14), were isolated from mix culture fermentation of the fungal endophyte *Fusarium tricinctum* and the bacterium *B. subtilis* 168 trpC2 on solid rice medium. *Fusarium tricinctum* was obtained from rhizomes of *Aristolochia paucinervis* of the mountains of Beni-Mellal, Morocco. Enniatins B1 **(193)** and A1 **(194)**, inhibit the growth the *B. subtilis* strain (MICs of 16 and 8 μ g/mL, respectively) and were also active against *S. aureus*, *S. pneumoniae*, and *E. faecalis* with MIC values in the range 2-8 μ g/mL. Lateropyrone **(192)** has antibacterial activity against *B. subtilis*, *S. aureus*, *S. pneumoniae* and *E. faecalis*, with MICs values ranging from 2 to 8 μ g/mL. All the above compounds were equally effective against a multi-drug-resistant clinical isolate of *S. aureus* (Ola et al., 2013).

Rhein **(195)** (Figure 14) was isolated from an endophyte *Fusarium solani* of *Rheum palmatum* collected at Ruoergai County, Sichuan Province, China. Rhein **(195)** naturally occurs in anthraquinone (1, 3, 8-trihydroxy-6-Me anthraquinone), that is found in *Rheum palmatum* L. and related plants such as rhubarb (You et al., 2013). It has good antibacterial activity with MICs in the range of 0.6–4 μg/mL against *S. aureus, S. aureus nor A, B. megaterium* 11561, *Pseudomonas syringae* and *Sinorhizobium meliloti* (Tegos et al., 2002).

Sanguinarine **(196)** (Figure 14), a benzophenanthridine alkaloid was obtained from the endophyte *Fusarium proliferatum* (strain BLH51) present on the leaves of *Macleaya cordata* of the Dabie Mountain, China. It has antibacterial, anthelmintic, and anti-inflammatory activities (Wang et al., 2014). It has antibacterial activities against the range of bacteria with MICs of 3.12–6.25 μg/mL against 15 clinical isolates of *S. aureus* while the MICs against of the two reference strains are 3.12 μg/mL for ATCC 25923 and 1.56 μg/mL for ATCC 33591.

The clinical isolates strains showed MIC values ranging from 31.25 to 250 μg/mL for ampicillin and 125–1000 μg/mL for ciprofloxacin. The treatment of the cells with sanguinarine induced the release of membrane-bound cell wall autolytic enzymes, which eventually resulted in lysis of the cell. Transmission electron microscopy (TEM) of MRSA treated with Sanguinarine show alterations in septa formation. The predisposition of lysis and altered morphology seen by TEM indicates that sanguinarine acts on the cytoplasmic membrane (Obiang-Obounou et al., 2011). The compound also has activity against plaque bacteria with MICs of 1–32 μg/mL for most species tested. The Electron microscopic studies of bacteria exposed to sanguinarine show that they aggregate and become morphologically irregular (Godowski, 1989).

Shikimic acid **(197)** (Figure 14), was obtained from the endophyte *Trichoderma ovalisporum* strain PRE-5 of the root of the herbal *Panax notoginseng.* The compound **(197)** is activity against *S. aureus*, *Bacillus cereus*, *M. luteus* and *E. coli* (Dang et al., 2010).

Trichoderic acid **(198)**, 2β-Hydroxytrichoacorenol **(199)**, Cyclonerodiol **(200)**, Cyclonerodiol oxide **(201)**, and Sorbicillin **(202)** (Figure 14), were isolated from a *Trichoderma* sp. PR-35, an endophyte of *Paeonia delavayi*. These compounds are active against *E. coli* and *S. albus* with minimal inhibitory amount (MIA) values in the range of 25–150 mg/disk. Compounds **(198)**, **(200)** and **(201)** are active against *Shigella sonnei* with MIA values in the range of 100–150 μg/disk (Wu et al., 2011).

Cyclopeptides PF1022F **(203)** and Halobacillin **(204)** (Figure 14), were isolated from the endophyte *Trichoderma asperellum* from traditional Chinese medicinal plant *Panax notoginseng*. Compounds **(203)** and **(204)** are active against *E. faecium* (CGMCC 1.2025) with IC_50_ values of 7.30 and 5.24 μ M and against *S. aureus* COL (CGMCC 1.2465) with IC_50_ values of 19.02 and 14.00 μ M, respectively (Ding et al., 2012).

**Figure 15:**
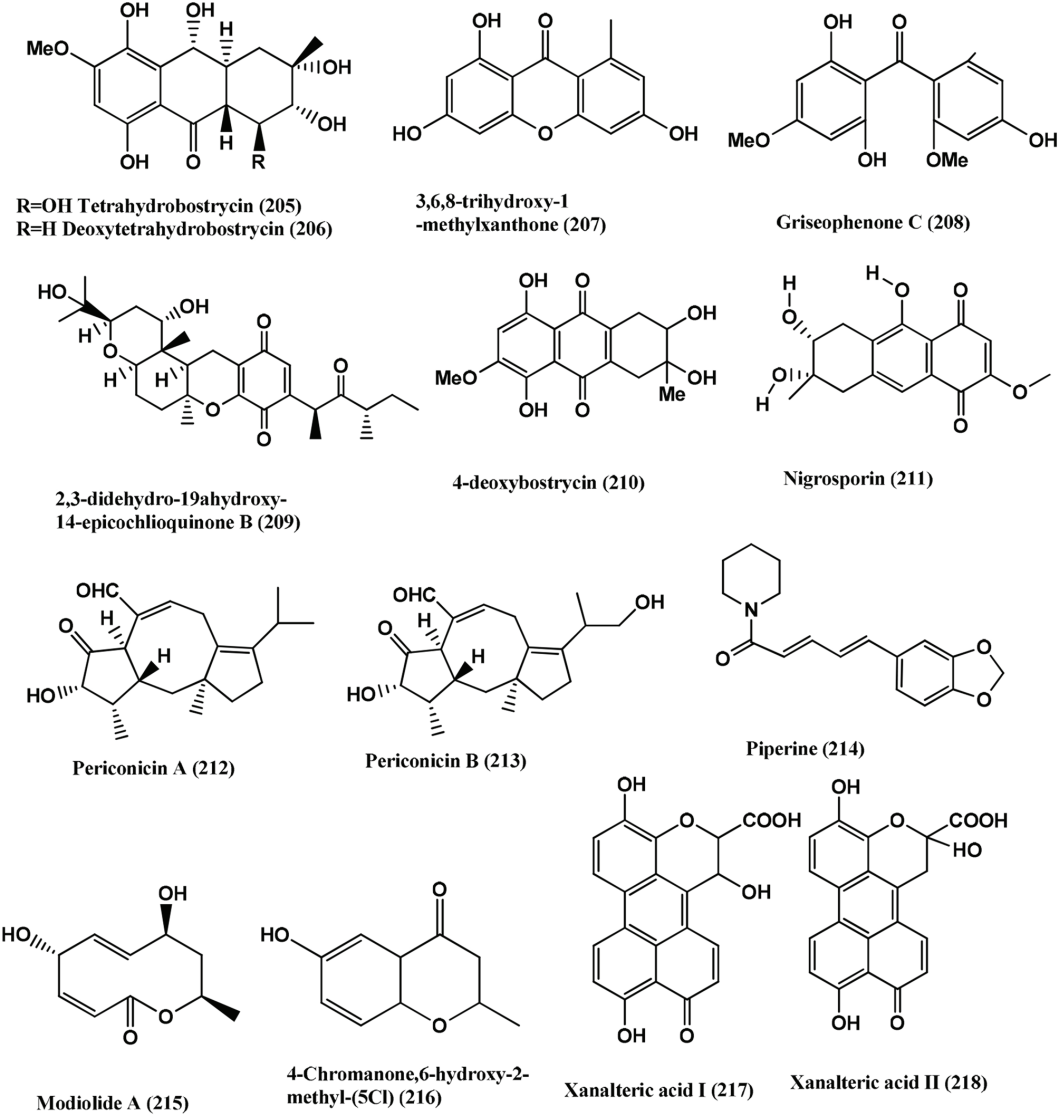
**Structures of antibacterial metabolites isolated from Hyphomycetes (205–218)**.

Tetrahydrobostrycin **(205)**, 4-Deoxytetrahydrobostrycin **(206)**, 3,6,8-Trihydroxy-1- methylxanthone **(207)**, Griseophenone C **(208)** and 2,3-Didehydro-19α-hydroxy-14-epicochlioquinone B **(209)** (Figure 15), were isolated from the endophyte *Nigrospora* sp. MA75, of the mangrove plant *Pongamia pinnata* collected from Guangxi Zhuang Autonomous Region of China. Compound **(209)** has excellent activity against all the tested bacteria (MRSA, *E. coli*, *P. aeruginosa*, *P. fluorescens* and *S. epidermidis*) with MIC values of 8, 4, 4, 0.5, and 0.5 μg/mL, respectively. The activity toward *E. coli*, *P. fluorescens* and *S. epidermidis* was stronger than that of the positive control (Ampicillin, with MICs values of 8, 4, and 4 μg/mL, respectively). Compound **(208)** strongly inhibits MRSA, *E. coli*, *P. aeruginosa*, and *P. fluorescens* at MIC values of 0.5, 2, 0.5, and 0.5 μg/mL, respectively. Compound **(205)** has significant activity toward MRSA and *E. coli* (MIC 2 and 0.5 μg/mL, respectively), while its analog compound **(206)**, is only activity against *E. coli* (MIC 4 μg/mL). This indicates that the OH group at C (4) could be important for the activity against MRSA. Compound **(207)** is active only against *S. epidermidis* (MIC 0.5 μg/mL) (Shang et al., 2012).

4-Deoxybostrycin **(210)** and its derivative Nigrosporin **(211)** (Figure 15), were isolated from the mangrove endophyte *Nigrospora* sp. of the South China Sea. These compounds are active against *M. tuberculosis* and clinical multidrug-resistant (MDR) *M. tuberculosis* strains with MIC values of <5–> 60 μ g/mL (Wang et al., 2013b).

Periconicins A **(212)** and B **(213)** (Figure 15), were isolated from an endophyte *Periconia* sp., from the branches of *Taxus cuspidata*. Periconicin A **(212)** has significant activity against *B. subtilis*, *S. aureus*, *K. pneumoniae*, and *Salmonella typhimurium* with MICs in the range of 3.12–12.5 μ g/mL. Periconicin B **(213)** has modest antibacterial activity against the same strains with MICs in the range 25–50 μ g/mL (Kim et al., 2004).

Piperine **(214)** (Figure 15), which was originally isolated from *Piper longum*, was also detected from the endophyte *Periconia* sp. of the same plant. Piperine has strong activity against *M. tuberculosis* and *M. smegmetis* with MICs of 1.74 and 2.62 μ g/mL respectively (Verma et al., 2011).

Modiolide A, 5, 8-dihydroxy-10-methyl-5, 8, 9, 10-tetrahydro-2H-Oxecin-2-one **(215)** and 4-Chromanone, 6-hydroxy-2-methyl- (5CI) **(216)** (Figure 15) were isolated from the endophyte *Periconia siamensis* (strain CMUGE015) of the leaves of the grass, *Thysanoleana latifolia* (Poaceae). Compound **(215)** is active against *Bacillus cereus*, *Listeria monocytogenes*, MRSA, *P. aeroginosa* and *E. coli* with MIC of 3.12, 6.25, 25.00, 12.50, and 50.00 μ g/mL respectively. Compound **(216)** is active against *B. cereus*, *Listeria monocytogenes*, MRSA, *P. aeruginosa* and *E. coli* with MICs of 6.25, 12.50, 50.00 25.00, 12.50, and 100.00 μ g/mL respectively (Bhilabutra et al., 2007).

**Figure 16:**
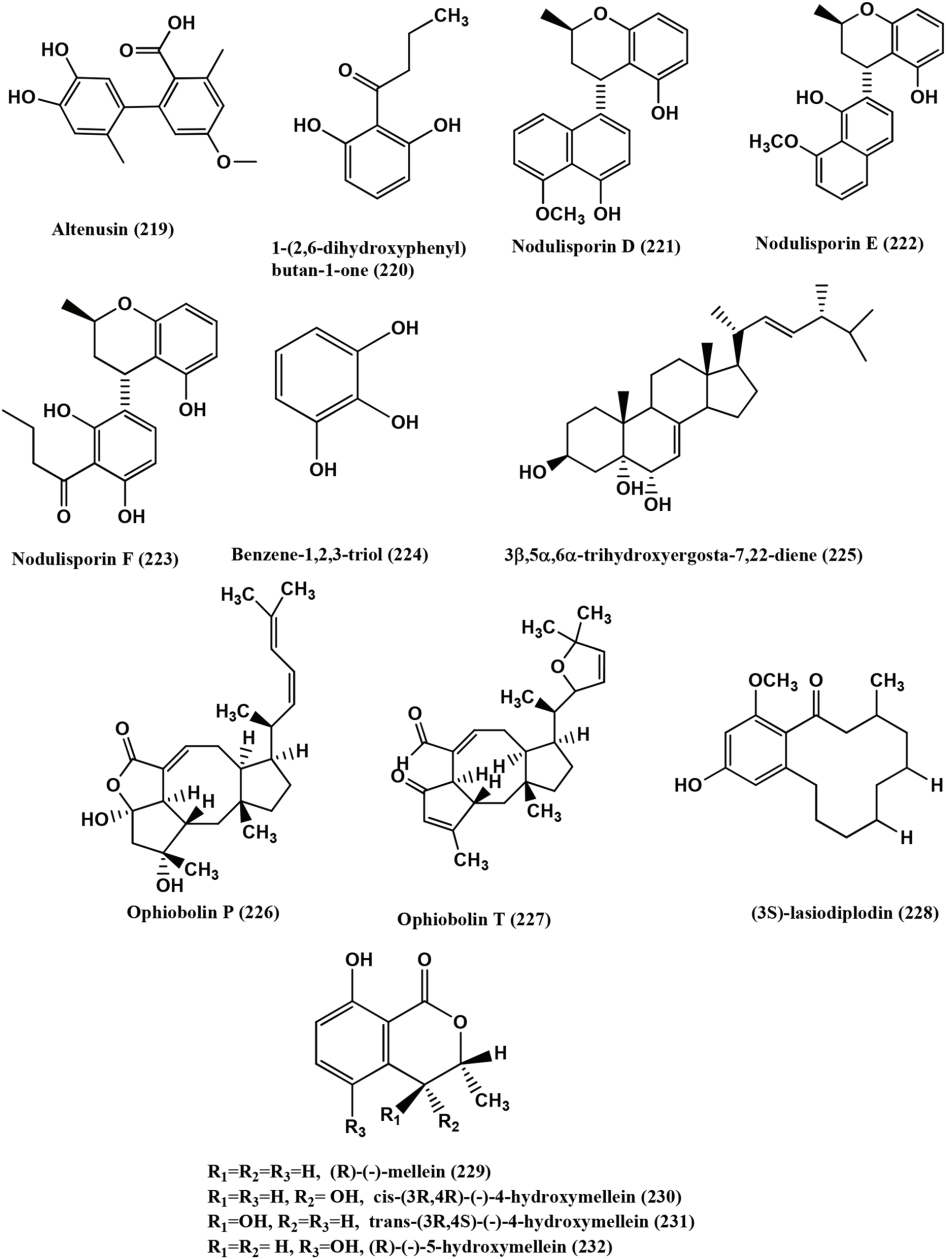
**Structures of antibacterial metabolites isolated from Hyphomycetes (219–232)**.

Xanalteric acids I **(217)** and II **(218)** (Figure 15) and Altenusin **(219)** (Figure 16), were obtained from *Alternaria* sp., of the mangrove plant *Sonneratia alba*. These **(217–218)** has weak antibacterial activities against MRSA with MICs of 125 and 250 μ g/mL. Altenusin **(219)** exhibited broad antimicrobial activity against several resistant pathogens (MRSA, *S. pneumonia, E. faecium, E. cloacae and A. faecalis*) with MIC values of 31.25–125 μ g/mL (Kjer et al., 2009).

1-(2, 6-dihydroxyphenyl) butan-1-one **(220)** (Figure 16), was isolated from the endophyte *Nodulisporium* sp. of *Juniperus cedrus* from Gomera Island. Compound **(220)** is active against *B. megaterium* at 0.25 mg/filter disc with 15 mm zone of inhibition (Dai et al., 2006).

Nodulisporins D-F **(221–223)**, Benzene- 1, 2, 3-triol **(224)** (Figure 16), were isolated from an endophyte *Nodulisporium* sp. of *Erica arborea*. Compounds **(221–224)** showed activity against *B. megaterium* (Dai et al., 2009b).

Pyrrocidine (**113**) (Figure 9), was isolated from *Acremonium zeae* an endophyte of maize. Compound **(113)** has potent activity against *Clavibacter michiganense* subsp. *Nebraskense* a causal agent of Goss's bacterial wilt of maize (MICs 1–2 μ g/mL), as well as *Bacillus mojavensis* (MICs 1–2 μ g/mL) and *P. fluorescens* (MICs 1–2 μ g/mL) (Wicklow and Poling, 2009).

Rhizoctonic acid **(139)**, Monomethylsulochrin **(138)** (Figure 9), Ergosterol **(79)** (Figure 5) and 3β, 5α, 6β-trihydroxyergosta-7, 22-diene **(225)** (Figure 16), were isolated from a *Rhizoctonia* sp. (Cy064), the endophyte in the leaves of *Cynodon dactylon*. Compounds **(139, 138, 79, and 225)** are active against five clinical and one reference strain of *H. pylori* (ATCC 43504) with MICs in the range 10.0–30.0 μ g/mL (Ma et al., 2004).

Ophiobolins P **(226)** and T **(227)** (Figure 16), were isolated from the endolichenic fungus *Ulocladium* sp. Ophiobolins P has moderate antibacterial activity against *B. subtilis* and MRSA with MICs of 62.5 and 31.3 μ g/mL respectively. Ophiobolin T **(227)** has moderate activity against *B. subtilis* and MRSA and Bacille Calmette-Guerin strain with MICs of 31.3 15.6 and 31.3 μ g/mL respectively (Wang et al., 2013a).

The antibacterial naphthaquinone Javanicin **(181)** (Figure 13) was isolated from an endophyte *Chloridium* sp. of *Azadirachta indica*. This compound is very active against *P. fluorescens* and *P. aeruginosa* with MIC of 2 μ g/mL (Khrawar et al., 2009).

(3S)-Lasiodiplodin **(228)**, (R)-(−)-Mellein **(229)**, Cis-(3R, 4R)-(−)-4-Hydroxymellein **(230)**, trans-(3R, 4S)-(−)-4-Hydroxymellein **(231)**, (R)-(−)-5-Hydroxymellein **(232)** (Figure 16) were isolated from the endophyte *Botryosphaeria rhodina* PSU-M35 and PSU-M114. Compound **(228)** is very active against *S. aureus* and MRSA with MIC values of 64 and 128 μ g/mL respectively. Compounds **(229–232)** have much weaker activities than compound **(228)** with MIC values >128 μ g/mL (Rukachaisirikul et al., 2009).

**Figure 17:**
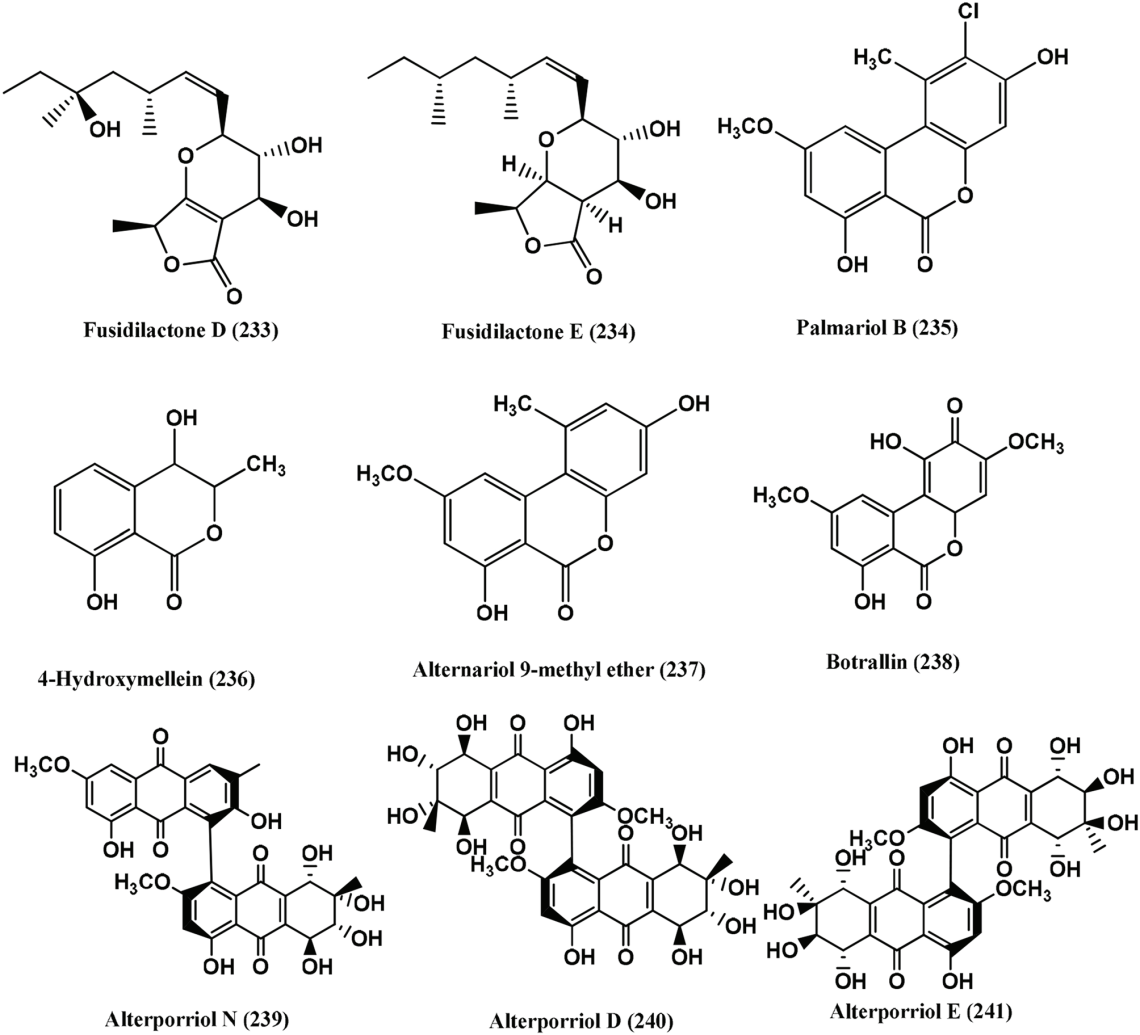
**Structures of antibacterial metabolites isolated from Hyphomycetes (233–241)**.

Fusidilactones D **(233)** and E **(234)** (Figure 17) were isolated from the endophyte, a *Fusidium* sp. from the leaves of *Mentha arvensis* growing in a meadow near Hahausen, Lower Saxony, Germany. Both compounds are weakly active against *E. coli* and *B. megaterium* (Qin et al., 2009).

Palmariol B **(235)**, 4-Hydroxymellein **(236)**, Alternariol 9-methyl ether **(237)** and Botrallin **(238)** (Figure 17) were isolated from an endophyte, *Hyalodendriella* sp. Ponipodef 12, of the hybrid “Neva” of *Populus deltoides* Marsh × *P. nigra* L. MIC_50_ values of the compounds on *Agrobacterium tumefaciens* ranged from 18.22 to 87.52 μ g/mL. Against *B. subtilis*, *P. lachrymans*, *R. solanacearum* and *X. vesicatoria*, MICs_50_ were from 19.22 to 98.47, 16.18 to 92.21, 16.24 to 85.46 and 17.81 to 86.32 μ g/mL respectively (Meng et al., 2012).

Alterporriol N **(239)**, Alterporriol D **(240)**, and Alterporriol E **(241)** (Figure 17), were isolated from *Stemphylium globuliferuman* an endophyte of *Mentha pulegium* collected from Morocco. Alterporriol N **(239)** is active against MRSA and *E. faecalis* with MICs of 62.5 and 15.63 μ g/mL. Alterporriol D **(240)** is active against MRSA and *Streptomyces pneumonia* with an MIC of 31.25 μ g/mL. Alterporriol E **(241)** is active against *S. pneumonia, E. faecalis* and *Enterobacter cloacae* with MICs of 31.25 μ g/mL each (Debbab et al., 2009).

### Compounds produced from unidentified fungi

**Figure 18:**
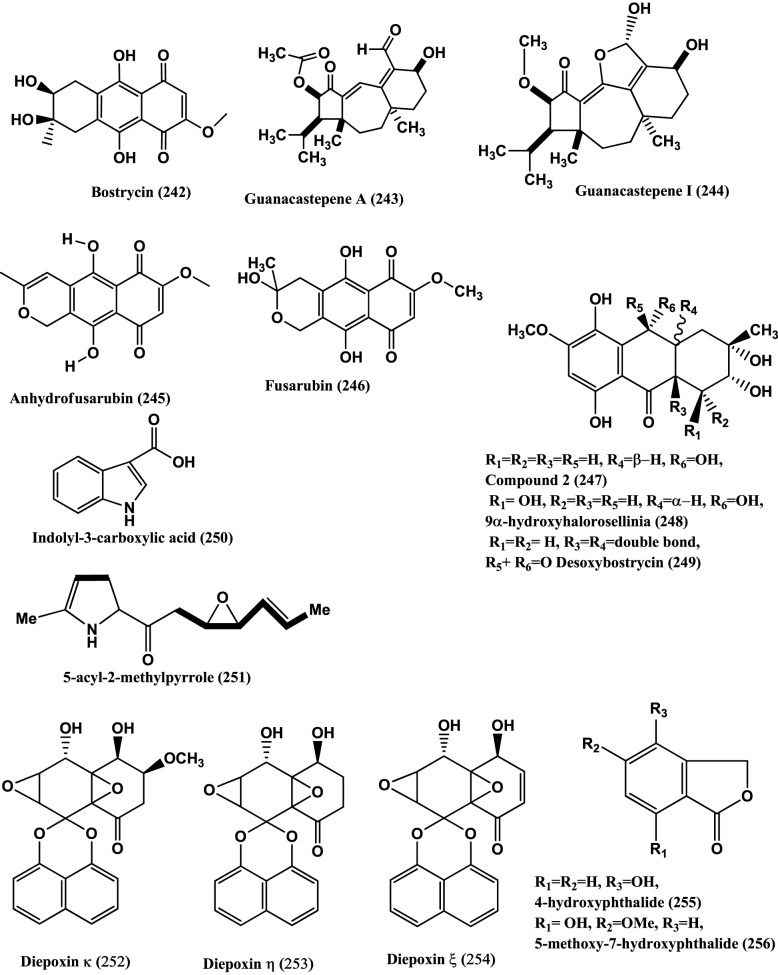
**Structures of antibacterial metabolites isolated from Unidentified fungus (242–256)**.

Nonsporulating fungi form a major group of such endophytes. Khafrefungin, Arundifungin are antifungals reported from such fungi (Deshmukh and Verekar, 2012). Bostrycin **(242)** (Figure 18) isolated from the mangrove endophyte, no. 1403, of the South China Sea (Xu et al., 2010a), shows antibacterial activity against *B. subtilis* (Charudattan and Rao, 1982).

Guanacastepene A **(243)** (Figure 18), a novel diterpenoid produced the fungus CR115 isolated from the branch of *Daphnopsis americana* growing in Guanacaste, Costa Rica, may prove to belong to potentially new class of antibacterial agents with activities against MRSA and VRE (Singh et al., 2000). Guanacastepene I **(244)** (Figure 18), was isolated from the same fungus is active against *S. aureus* (Brady et al., 2001).

Anhydrofusarubin **(245)** (Figure 18), was isolated from the endophyte no. B77 of a mangrove tree on the South China Sea coast. Compound (**245)** is active against *Staphylococcus aureus* (ATCC27154) with a MIC of 12.5 μ g/mL (Shao et al., 2008b).

3-O-Methylfusarubin **(182)** (Figure 13), Fusarubin **(246)** (Figure 18), were isolated from the endophyte B77 present in the seeds of the mangrove plant *Kandelia candel* in Zhanjiang. Compounds **(182)** and **(246)** were active against *S. aureus* ATCC 27154 with MIC values of 50.0 and 12.5 μ g/mL, respectively (Shao et al., 2008a).

Compound **(247)**, 9α –Hydroxyhalorosellinia A **(248)** and Desoxybostrycin **(249)** (Figure 18), were isolated from the endophyte PSU-N24 present in the plant *Garcinia nigrolineata* collected from the Ton Nga Chang wildlife sanctuary, Songkhla province, southern Thailand. Compound **(248)** was active against *M. tuberculosis* with the MIC value of 12.50 μ g/mL whilst compounds **(247)** and **(249)** had MIC values of 25 and 50 μ g/mL, respectively (Sommart et al., 2008).

Indolyl-3-carboxylic acid **(250)** (Figure 18), isolated from the endophyte S20 of *Cephalotaxus hainanensis* Li. showed inhibition of *S. aureus* and MRSA with diameters of inhibition zones of which were 12 and 8 mm, respectively when 50 μ l of the compound (10 mg/mL) impregnated on sterile filter paper discs (6-mm diameter) (Dai et al., 2009a). The structure of a new 5-acyl-2-methylpyrrole **(251)** (Figure 18) from the same endophyte S20 of *Cephalotaxus hainanensis*, was shown to be 1-(5-methyl-1H-pyrrol-2-yl)-2-((2S*, 3R*)-3-((E)-prop-1-enyl) oxiran-2-yl) ethanone. Compound **(251)** is active against *S. aureus* and MRSA. The diameters of inhibition are 12.0 mm and 10.0 mm respectively when 50 μ L (10 mg/mL) of the compound was impregnated on sterile filter paper discs (6-mm diameter) (Dai et al., 2009c).

Spirobisnaphthalenes, namely Diepoxin κ **(252)**, Diepoxin η **(253)**, and Diepoxin ζ **(254)** (Figure 18), were isolated from the endophyte Dzf12 of the medicinal plant *Dioscorea zingiberensis*. Among these, compound **(252)** has antibacterial activity, against *E. coli*, *A. tumefaciens, X. vesicatoria, P. lachrymans* and *B. subtilis* with MICs from 50 to 100 μ g/mL. A mixture of diepoxin η **(253)**, and diepoxin ζ **(254)** showed antibacterial activity against the same set of bacteria with a MICs range of 5.0–12.5 μ g/mL (Cai et al., 2009).

**Figure 19:**
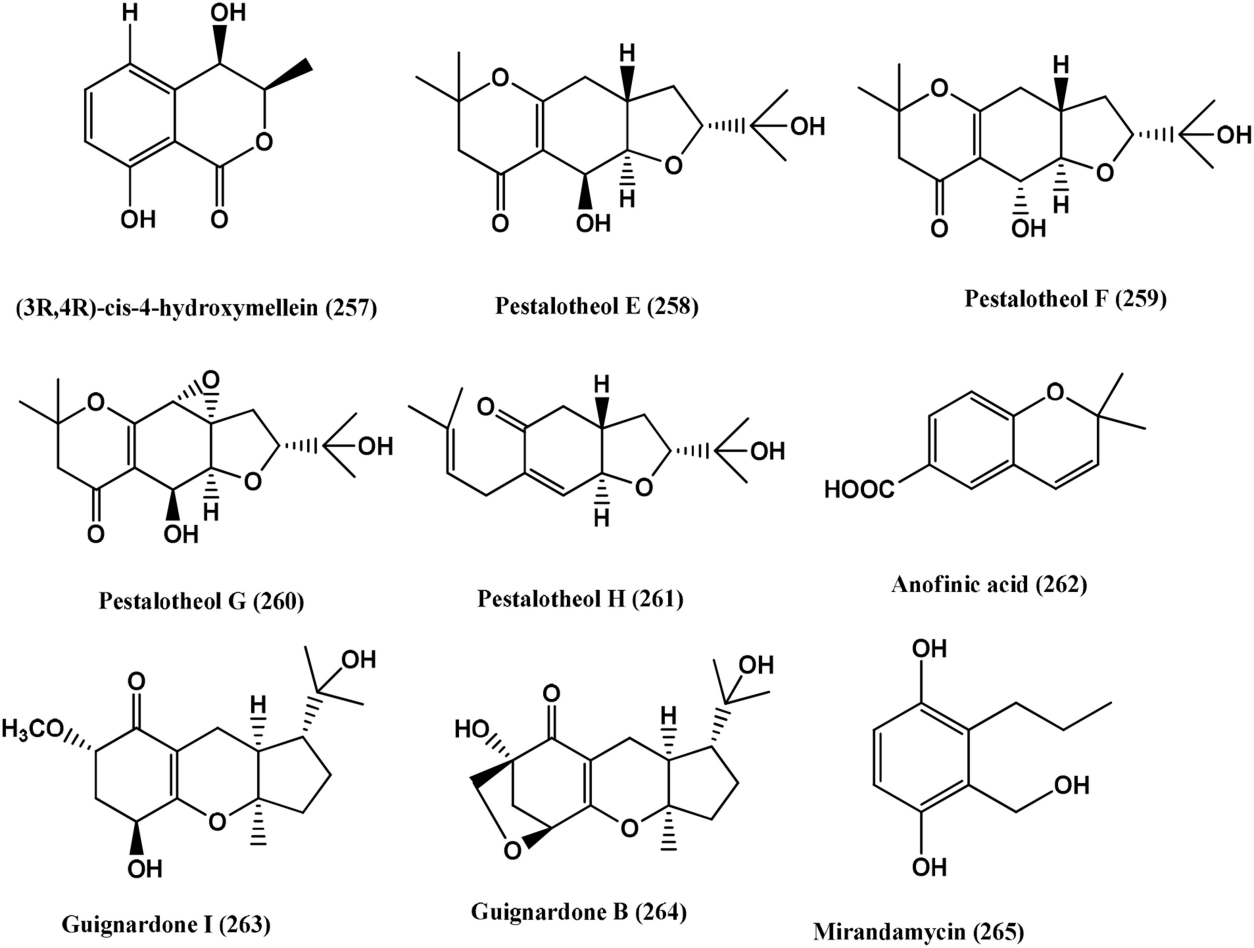
**Structures of antibacterial metabolites isolated from Unidentified fungus (257–265)**.

4-Hydroxyphthalide **(255)**, 5-methoxy-7-hydroxyphthalide **(256)**, (3R, 4R)-cis-4 hydroxymellein **(257)** (Figure 19), were obtained from an unidentified Ascomycete from *Meliotus dentatus* of the coastal area of the Baltic Sea, Ahrenshoop, Germany. Compounds **(255)** and **(256)** were active against *E. coli* whereas **(256)** and **(257)** were active against *B. megaterium* (Hussain et al., 2009b).

Pestalotheols E-H **(258–261)** and Anofinic acid **(262)** (Figure 19), were obtained from an unidentified ascomycete of *Arbutus unedo*. Compounds **(258–262)** have antibacterial activity against *E. coli* and *B. megaterium* (Qin et al., 2011).

Guignardone I **(263)** and Guignardone B **(264)** (Figure 19), were isolated from an endophyte fungus A1 of the mangrove plant *Scyphiphora hydrophyllacea*. Guignardone I **(263)** shows zones inhibition of 9.0 and 11.0 mm in diameter, using 6 mm filter paper discs toward MRSA and *S. aureus* at 65 μ M, respectively. Guignardone B **(264)** shows zones of 8.0 mm against MRSA at 65 μ M. Kanamycin sulfate, used as positive control (10 μ L of 0.08 mg/mL) showed an inhibition zone of 30 mm (Mei et al., 2012).

Mirandamycin **(265)** (Figure 19) was obtained from isolate 1223-D, an unclassified fungus of twig of *Neomirandea angularis* of family Asteraceae. It is active against *E.coli* 25922, *P. aeruginosa* 27853, *K. pneumoniae* carbapenemase positive BAA-1705, MRSA BAA-976 and *V. cholerae* PW357 with MICs of 80, 80, >80, 10, and 40 μg/mL respectively (Ymele-Leki et al., 2012).

#### Volatile organic compounds from endophytic fungi

Strobel et al. (2001) reported at least 28 volatile organic compounds (VOC) from the xylariaceaous endophyte *Muscodor albus* (isolate 620), of *Cinnamomum zeylanicum* from Lancetilla Botanical Garden near La Ceiba, Honduras. These VOC's are mixtures of gasses of five class's viz. alcohols, acids, esters, ketones and lipids. The most effective were the esters, of which, 1-butanol, 3-methyl-acetate has the highest activity. The VOC's inhibited and killed certain bacteria, within a period of 1–3 days. Most test organisms were completely inhibited, and in fact killed. These includes *Escherichia coli, Staphylococcus aureus, Micrococcus luteus* and *Bacillus subtilis* along with some fungal species.

Strain of *Muscodor* namely *Muscodor crispans* of *Ananas ananassoides* (wild pineapple) growing in the Bolivian Amazon Basin produces VOC's; namely propanoic acid, 2-methyl-; 1-butanol, 3-methyl-; 1-butanol, 3-methyl-, acetate; propanoic acid, 2-methyl-, 2-methylbutyl ester; and ethanol. The VOC's of this fungus are effective against *Xanthomonas axonopodis* pv. *citri* a citrus pathogens. The VOC's of *M. crispans* kill several human pathogens, including *Yersinia pestis*, *Mycobacterium tuberculosis* and *Staphylococcus aureus*. *Muscodor crispans* is only effective against the vegetative cells of *Bacillus anthracis*, but not against the spores. Artificial mixtures of the fungal VOC's were both inhibitory and lethal to a number of human and plant pathogens, including three drug-resistant strains of *Mycobacterium tuberculosis* (Mitchell et al., 2010). The mechanism of action of the VOC's of *Muscodor* spp. on target bacteria is unknown. A microarray study of the transcriptional response analysis of *B. subtilis* cells exposed to *M. albus* VOC's show that the expression of genes involved in DNA repair and replication increased, suggesting that VOC's induce some type of DNA damage in cells, possibly through the effect of one of the naphthalene derivatives (Mitchell et al., 2010).

#### Outlook

A definite, urgent and worldwide effort is needed to tackle the problems of the populations in third world and developing countries. MRSA, VRE, PRSP, ESCAPE organisms have spread through these countries over the years particularly due to immunocompromised populations. *Mycobacterium tuberculosis* is a major threat! and New and Novel drugs are a must!! Endophytic fungi may be an excellent source of such compounds. These organisms have a vast repertoire of diverse chemicals such as steroids, xanthones, phenols, isocoumarins, perylene derivatives, quinones, furandiones, terpenoids, depsipeptides and cytochalasins (Tan and Zou, 2001; Gunatilaka, 2006; Zhang et al., 2006; Guo et al., 2008).

A major challenge in Drug Discovery Program based on endophytic fungi lies in developing effective strategies to isolating bioactive strains. Strobel and Daisy (2003) suggested that areas of high biodiversity of endemic plant species may hold the greatest potential for endophytes with novel chemical entities. Tropical forests are some of the most bio diverse ecosystems and their leaves are “biodiversity hotspots” (Arnold and Lutzoni, 2007). The selection of plants is crucial. Those with medicinal properties should be given preference. Metabolites produced by fungi need to correlated with the plant genomics, thus allowing far better knowledge of biosynthetic pathways. This will also justify the production of metabolites rather than unproven hypotheses.

Identification of endophytic fungi using molecular analyses provides an opportunity to look for broad patterns in bioactivity not only at the genotype or strain level, but at higher taxonomic levels that may in turn assist in focusing on the association of metabolite with the plant.

The endophytic flora of the Indian subcontinent has been explored for their diversity but not enough for their bioactive metabolites. The published work is scanty (Puri et al., 2005; Deshmukh et al., 2009; Khrawar et al., 2009; Periyasamy et al., 2014). There is a need for groups from different scientific discipline (mycologist, chemist, toxicologist, and pharmacologist) to engage in this search process. Enormous natural wealth exists in the world's tropical forests, but disparity exists between developed countries with their financial resources and biodiversity rich countries with underdeveloped economy and limited funds. May be funding agencies need to look at such aspects.

The need of a more and larger collection of fungal endopytes is suggested. Bioactive metabolite metabolites from such collections could yield leads for pharmaceutical and agricultural application.

What emerges is the essential bonding of various discipline of biology and chemistry into cohesive target delivery vehicles.

### Conflict of interest statement

The authors declare that the research was conducted in the absence of any commercial or financial relationships that could be construed as a potential conflict of interest.
